# Ultrathin Zincophilic Interphase Regulated Electric Double Layer Enabling Highly Stable Aqueous Zinc-Ion Batteries

**DOI:** 10.1007/s40820-023-01312-1

**Published:** 2024-01-25

**Authors:** Yimei Chen, Zhiping Deng, Yongxiang Sun, Yue Li, Hao Zhang, Ge Li, Hongbo Zeng, Xiaolei Wang

**Affiliations:** 1 Department of Chemical and Materials Engineering, University of Alberta, 9211-116 Street NW, Edmonton, AB T6G 1H9 Canada; 2 Department of Mechanical Engineering, University of Alberta, 9211-116 Street NW, Edmonton, AB T6G 1H9 Canada

**Keywords:** Zinc anode, Electric double-layer regulation, Multifunction SEI layer, Inhibited side reactions and dendrite, Rapid kinetics

## Abstract

The practical application of aqueous zinc-ion batteries for large-grid scale systems is still hindered by uncontrolled zinc dendrite and side reactions. Regulating the electrical double layer via the electrode/electrolyte interface layer is an effective strategy to improve the stability of Zn anodes. Herein, we report an ultrathin zincophilic ZnS layer as a model regulator. At a given cycling current, the cell with Zn@ZnS electrode displays a lower potential drop over the Helmholtz layer (stern layer) and a suppressed diffuse layer, indicating the regulated charge distribution and decreased electric double layer repulsion force. Boosted zinc adsorption sites are also expected as proved by the enhanced electric double-layer capacitance. Consequently, the symmetric cell with the ZnS protection layer can stably cycle for around 3,000 h at 1 mA cm^−2^ with a lower overpotential of 25 mV. When coupled with an I_2_/AC cathode, the cell demonstrates a high rate performance of 160 mAh g^−1^ at 0.1 A g^−1^ and long cycling stability of over 10,000 cycles at 10 A g^−1^. The Zn||MnO_2_ also sustains both high capacity and long cycling stability of 130 mAh g^−1^ after 1,200 cycles at 0.5 A g^−1^.

**Supplementary Information:**

The online version contains supplementary material available at 10.1007/s40820-023-01312-1.

## Highlights

- Electric double-layer regulation enabled by an ultrathin multifunctional solid electrolyte interphase layer with zincophilicity and rapid transport kinetics.
- Lowered potential drop over the Helmholtz layer and suppressed diffuse layer.
- Inhibited side reactions and uniform zinc deposition.

**Supplementary Information:**

The online version contains supplementary material available at 10.1007/s40820-023-01312-1.

## Graphical abstract

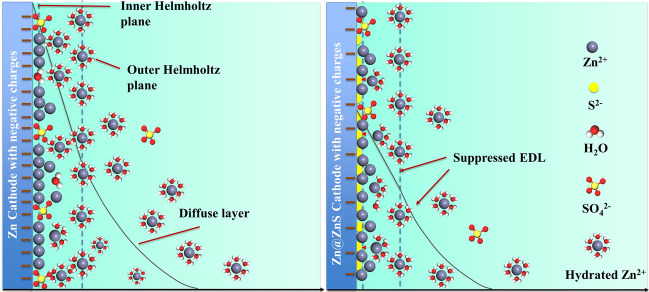

## Introduction

Aqueous zinc-ion batteries (AZIBs), featuring low cost, high safety, and satisfactory theoretical capacity (820 mAh g^–1^/5855 mAh cm^–2^), can be a powerful candidate in future grid-scale energy storage [1–4]. However, the uncontrolled zinc dendrite and parasitic side reactions still hinder the development and practical application of AZIBs [1–3]. The aforementioned issues are strongly related to the electric double-layer (EDL) structure since the electrochemical reaction happens in this minuscule region [1]. Specifically, during the charging process, the hydrated zinc ions must first dissociate from the water molecules at the Helmholtz layer before undergoing reduction [1]. This will leave rich H_2_O on the EDL and hydrate the electrode surface, inducing corrosion and hydrogen evolution (HER) [2]. Besides, under the EDL repulsion force, the Zn^2+^ has the propensity to deposit at protrusions induced by the inhomogeneous electric field, leading to the formation of a loose deposition layer [1].

Tremendous efforts have been devoted to solving these issues, such as designing eutectic electrolytes or highly concentrated systems [2–7] and introducing electrolyte additives [8–13]. However, the efficacy of these strategies greatly depends on the additive utilized. Chen’s group points out that for electrolyte additives, the ability to form a solid electrolyte interphase (SEI) other than parameters of adsorption energy, donor numbers, and dielectric constant, predominates the EDL structure and influences the anode stability [14]. Based on this finding, it is expected that building an SEI will endow uniform zinc flux and deposition.

In theory, three key factors should be considered for choosing potential SEI candidates [15]. Firstly, high Zn^2+^ conductivity and electron insulation are required to lower the concentration polarization and inhibit hydrogen reduction. Secondly, materials should be tensile enough and show good adherence to the substrate, enabling their stable structure and tolerance to drastic volume changes. Finally, a thin and dense structure is desired to isolate the electrolyte and reduce unnecessary weight caused by thick coatings. Generally, the in situ formed SEI layer by electrolyte decomposition upon cycling [16–18] which concerns the continuous consumption of electrolytes and uncontrollable thickness, is not desirable for battery cycling. As a result, building an artificial SEI layer is a promising alternative. Various metal oxides like CaCO_3_, TiO_2_, and Fe_2_O_3_ have been studied as coating materials for anode protection, but poor adhesion and stretchability can cause cracks and the detachment of the coatings from the electrode [19–24]. Metal nanoparticles, such as Au, and Ag with high overpotential for Zn^2+^ reduction, are not suitable for hydrogen evolution (HER) suppression [25–27]. Currently, some zincophilic semiconductors with electronegative elements, like ZnF_2_, ZnO, ZnS, and ZnSe, have received a popular application in electrode engineering due to their strong affinity to Zn^2+^ and lowered nucleation barriers [16, 28–31]. However, most of these reported SEI layers are synthesized under stringent conditions, such as a high vacuum or low oxygen-deprived atmosphere at high temperatures, which challenges mass production [28, 30]. Additionally, the diffusion of the Zn^2+^ through the SEI layer must overcome some energy barriers, making it necessary to control the thickness to minimize the diffusion path.

**Scheme 1:**
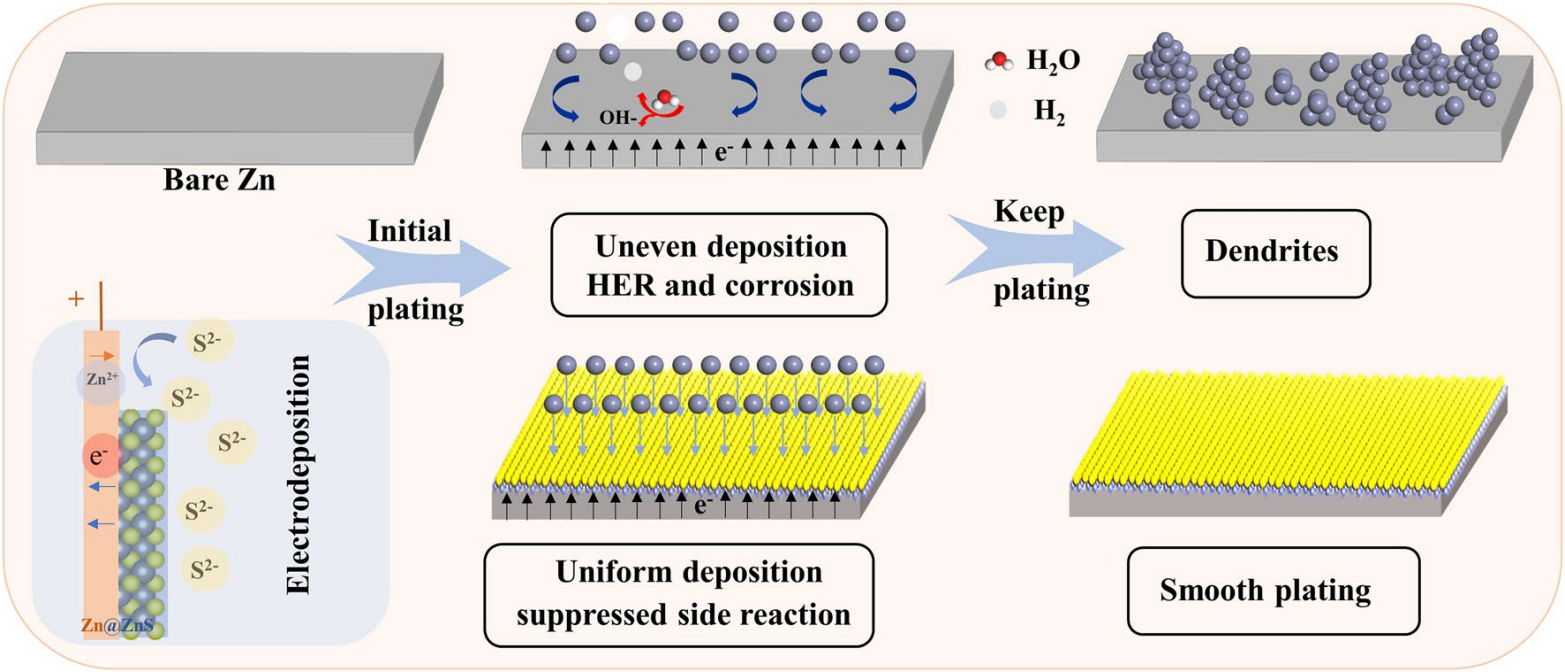
Schematic of zinc deposition process at the Zn and Zn@ZnS surface

Herein, an ultrathin nanoparticle-like ZnS SEI layer is selected as a model EDL regulator. As shown in Scheme 1, the zincophilic ZnS layer not only cuts off the electron transfer, eradicating water-induced parasitic reactions but also redistributes the concentrated electric field and electrolyte current density [32, 33]. The protective layer also provides more active sites for Zn^2+^ adsorption. As a result, a dendrite-free and side reactions suppression performance could be expected. More encouragingly, both the simulation and electrochemical characterizations confirmed that the presence of the SEI layer regulates charge distribution and decreases EDL repulsion force, and induces a compact and dense zinc deposition. With the optimized EDL, the Zn@ZnS symmetric cells sustain ultra-stable cycling of nearly 3,000 h at 1 mA cm^−2^, accompanied by decreased voltage hysteresis (43.6 mV). A high average reversibility of 98.9% is obtained in 2,500 cycles at a high current density of 5 mA cm^−2^. This work highlights the importance of EDL regulation on the behavior of zinc electrodeposition.

## Results and Discussion

### Construction, Morphology, and Properties of ZnS Layer

**Fig. 1:**
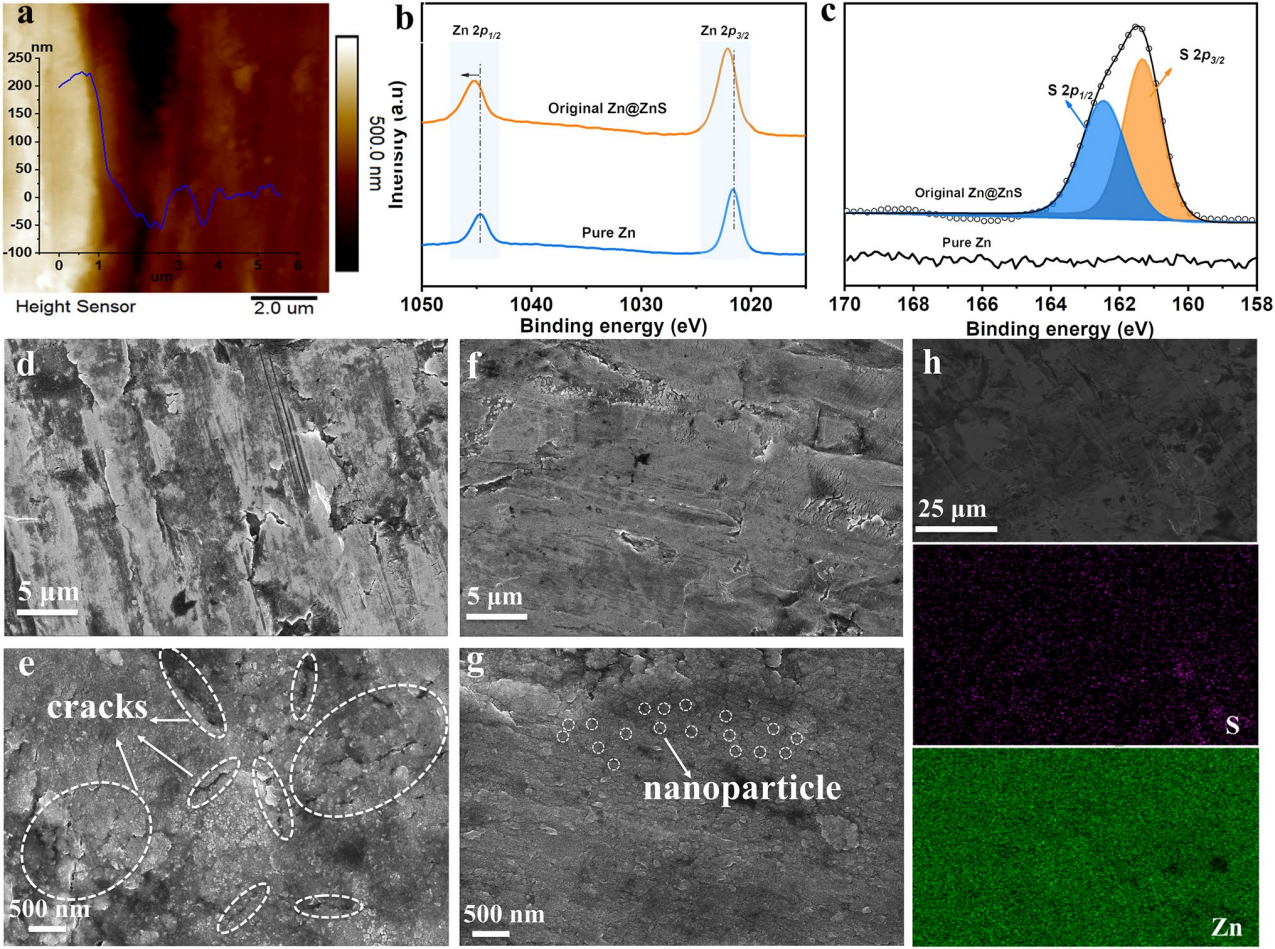
Morphology and properties of ZnS Layer. **a** Atomic force microscopy image of the electrode (right part: without ZnS; left part: with ZnS deposition). **b, c** XPS of Zn and Zn@ZnS electrodes. **d, e** SEM images of pure Zn electrode. **f, g** SEM images of Zn@ZnS electrode. **h** EDX- mapping of Zn@ZnS surface

The ultrathin ZnS layer was galvanostatically deposited on the zinc surface in 0.2 M Na_2_S solution. Upon the current flow (2.5 mA cm^−2^), the zinc metal underwent oxidation and released Zn^2+^, which then reacted with S^2−^ in the inner Helmholtz plane (IHP), forming the uniform ZnS SEI layer with a thickness of 200 nm over an electrodeposition time of 2 min (Figs. 1a and S1). It should be noted that the thickness is in the same order but lower than the theoretical value of 380 nm due to the lower current efficiency [34]. Importantly, the ZnS SEI layer demonstrates a strong adhesion to the Zn substrate, and even after being twisted, no cracks are observed on the ZnS surface (Fig. S2). The chemical composition of the ZnS film was analyzed by X-ray photoelectron spectroscopy (XPS). Figures 1b, c and S3 indicate the pristine Zn foil only presents the Zn 2*p*_1/2_ and Zn 2*p*_3/2_ signal with the binding energies of 1045 and 1022 eV, respectively [35]. In contrast, the Zn@ZnS electrode exhibits distinct S 2*p*_1/2_ (162.4 eV) and S 2*p*_3/2_ (161.3 eV) peaks, indicating the successful synthesis of ZnS [28]. Besides, the binding energy of zinc in the Zn@ZnS electrode shifts to a higher value due to the formation of Zn–S bonds. Good wettability of the electrolyte to the electrode is a crucial factor in optimizing the zinc nucleation behavior by advancing the Zn^2+^ diffusion toward the electrode and decreasing the nucleation energy (Eqs. S16 and S17) while retaining the nucleation radius [16, 36]. Figure S4 shows the contact angle for the Zn@ZnS electrode (71°) is smaller, compared to the bare Zn electrode (106.5°), confirming a better wettability of the electrolyte toward Zn@ZnS electrode due to the stronger interaction with ZnS.

The morphology evolution before and after the ZnS SEI layer was studied by the scanning electron microscope (SEM) images. As depicted in Fig. 1d, e, the pure Zn surface displays numerous scratches and cracks due to its rough manufacturing. These imperfections induce the “tip effects” that unevenly distributed electric field aggravates the uniform deposition of Zn. With the ultrathin ZnS SEI layer (Fig. 1f, g), the electrode shows a flatter and smoother surface, and nanoparticle-like morphology can be observed. Such structure can increase the contact area of the electrode/electrolyte and guide the uniform diffusion and deposition of Zn. The atomic force microscope (AFM) images also suggest a smoother surface with the ZnS SEI layer where the surface average roughness (*R*_*a*_) decreased from 24.3 nm for the bare Zn surface to 11.9 nm for ZnS covered surface (Fig. S5). In addition, the energy-dispersive X-ray spectroscopy (EDX) mapping images in Fig. 1h verified that S and Zn elements are homogeneously distributed across the surface, confirming the uniformity of the ZnS protection layer. The cross-sectional EDX mapping results shown in Fig. S6 also display the distribution of ZnS on the surface of the Zn electrode with an approximate thickness of 200 nm. The XRD results of the prepared Zn@ZnS electrode surface show no extra peaks except the Zn metal signal (Fig. S7). That may be due to the amorphous state of deposited ZnS. The thickness and morphology of this ZnS layer can be adjusted by controlling the electrodeposition current and time. Lower current density may cause the poor coating or high impurity of the deposits while higher current density tends to result in the severe agglomeration of deposits [37–39]. Besides, at a given capacity, a lower electrodeposition current generally corresponds to a thinner coating thickness due to the low current efficiency [37, 38]. The SEM images in Fig. S8 exhibit that at a low current density of 1.25 mA cm^−2^_,_ cracks are still obvious on the surface, while with a high current density of 5 mA cm^−2^, small nanoparticles appear on the surface. However, these particles are not uniform and tend to aggregate in some areas, which is consistent with the phenomenon observed in previous reports.

### Regulated Electric Double Layer

As stated by Derjaguin–Landau–Verwey–Overbeek (DLVO) theory, the zinc ions in aqueous solutions suffer the electric repulsive force brought from the counterions in EDL as well as the van der Waals attractive force (VDW) [40]. Under the repulsive governed force, the zinc ions tend to deposit at “tips” through a 2D diffusion and form a loose structure, whereas a compact and dense structure can be obtained with attractive force. In this regard, a regulated EDL repulsion force is desired. According to the Poisson–Boltzmann (PB) equation, the reduced EDL repulsive force can be reflected in the suppressed EDL thickness, which can be described by Debye length [40].

**Fig. 2:**
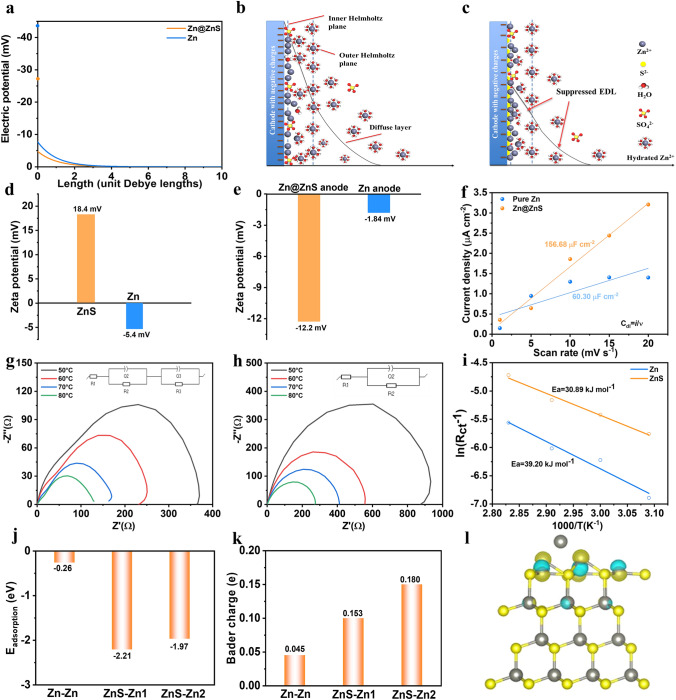
Simulations of Zn and ZnS properties. **a** The potential difference between the blue dot (yellow dot) and the value of the blue line (yellow line) at x = 0 represents the potential difference over the Helmholtz layer. **b, c** Scheme of EDL structure of **b** Zn/electrolyte and **c** Zn@ZnS/electrolyte interphase. **d** Zeta potential of zinc powder and ZnS powder. **e** Zeta potential of zinc deposits from Zn and Zn@ZnS surface. **f** EDL capacitance of Zn and Zn@ZnS symmetric batteries. **g, h** EIS of **g** Zn@ZnS||Zn@ZnS and **h** pure Zn||Zn symmetric cell at various temperatures. **i** The activation energy (*E*_*a*_) of Zn and Zn@ZnS symmetric cell. **j** Adsorption energy, **k** Bader charge, and **l** electron density difference of Zn adatom toward Zn or Zn@ZnS electrode (For charge density difference, the blue color indicates a decrease in charge, and yellow represents an increase in charge)

To reveal the suppression of EDL by the presence of the ZnS SEI layer, multiple calculations were performed. Firstly, the EDL structure with and without the ZnS SEI layer was modeled by COMSOL Multiphysics based on the experimental data. As shown in Fig. 2a, the potential difference over the Helmholtz layer is higher (36.4 mV) for the Zn electrode than the Zn@ZnS electrode (22 mV), demonstrating that Zn^2+^ suffers a lower energy barrier for the desolvation and reduction in the presence of ZnS layer. This weakened EDL repulsive force is conducive to dense zinc deposition. The cell with Zn@ZnS electrode also shows a suppressed diffuse layer, indicating a reduced EDL repulsive force between the charged particles. The reduced EDL repulsive force can reduce the diffusion barrier for the hydrated zinc ions. Figure 2b, c intuitively displays the composition of EDL without and with the ZnS SEI layer. The hydrated zinc ions, along with SO_4_^2−^ anions, can only approach the outer Helmholtz layer (OHL) [1, 14]. Subsequently, the accompanied SO_4_^2−^ anions, de-solvated Zn^2+^, and water molecules taken off from hydrates diffuse toward the zinc surface through the inner Helmholtz layer (IHL). With a bare Zn electrode, SO_4_^2−^ anions and water molecules can specifically absorb on the surface and cover the active site for Zn^2+^ adsorption, leading to side reactions, and the formation of the by-product. Besides, under the high repulsion force, Zn^2+^ tends to diffuse and deposit at the protrusions due to the “tip effects”, forming scattered platelets and potentially inducing zinc dendrite growth (Fig. 2b). With ZnS film, the tips were covered and a uniform surface with a strong affinity to the Zn^2+^ can be obtained (Fig. 2c). The enhanced desolvation effects via the strong interaction between S and adatom could exclude water molecules from entering the IHP and prevent the direct contact of water and Zn substrate, forming a water-poor environment [14].

To experimentally study the charge state of zinc anode in an aqueous solution, the zeta potential was tested. The zeta potential of zinc powder in aqueous solutions exhibits a negative value, suggesting the zinc surface is negatively charged (Fig. 2d) [41, 42]. By contrast, the zeta potential of ZnS powder is positive, with a high average value of 18.4 mV, consistent with the reported value [43]. The high zeta potential material is conducive to enhancing the electrokinetic effects of the battery by fastening the mass transport rate and adjusting ion distribution, which can be evidenced by the ions’ concentration gradient in the electrolyte (Fig. S9). Figure 2e shows the zeta potential of zinc deposits in pure Zn and Zn@ZnS surface. The results indicate both deposits present negative values, but a high absolute value of 12.2 mV for Zn@ZnS suggests the deposit is more stable from aggregating into a large nucleus, leading to uniform zinc deposition. The impact of the ZnS SEI layer on the EDL capacitance evolution was also studied by CV tests. Based on the equation *C* = ε*A*/*d*, where ɛ is the dielectric constant of the electrolyte,* A* is electrode surface area and *d* is the EDL thickness, higher capacitance can be obtained with a higher specific surface area and smaller EDL thickness. As shown in Figs. S10 and 2f, the symmetric cell with Zn@ZnS electrodes shows much higher capacitance (156.68 μF cm^−2^) than that using bare Zn electrodes (60.30 μF cm^−2^), which is attributed to the abundant deposition sites on Zn@ZnS surface and decreased electric double layer. The higher capacitance is conducive to homogeneous zinc diffusion and deposition [1]. The enhanced electrokinetic and rich deposition sites can increase the reaction rate and decrease the reaction barrier, thus decreasing the potential drop over the compact layer and suppressing the diffuse layer.

It is well known that the solvation structure dominated by water is significant in determining the reaction kinetics since the hydrated Zn^2+^ needs to be de-solvated before gaining electrons [44]. The activation energy (*E*_*a*_) is calculated based on the electrochemical impedance spectroscopy (EIS) tests. Figure 2g, h shows that with temperatures ranging from 50 to 80 ºC, the resistance of both electrodes decreases, and at a given temperature, the *R*_*ct*_ of the Zn@ZnS electrode (Fig. 2g) is much lower than that of the pure Zn anode (Fig. 2h), demonstrating an enhanced charge transfer capability. A semi-circle in a high-frequency area is also observed for the Zn@ZnS electrode, corresponding to the resistance associated with Zn^2+^ crossing the SEI layer. The calculated activation energy (*E*_*a*_) based on the Arrhenius equation and the fitting results (Table S2) for the Zn electrode is 39.20 kJ mol^−1^, in comparison to 30.89 kJ mol^−1^ for the ZnS SEI layer (Fig. 2i). The decreased activation energy for Zn@ZnS cell can be attributed to the strong interaction between Zn^2+^ and electronegative sulfur elements weakens the repulsive force. What’s more, the water molecules in the solvation sheath are being attracted by the ZnS layer when diffused toward the zinc surface, advancing the desolvation process and the charge transfer rate. Additionally, the Zn^2+^ transference number *tzn*^*2*+^ was studied to illustrate the Zn^2+^ conductivity in the ZnS SEI layer (Fig. S11). The symmetric cell with bare Zn electrodes shows a low *tzn*^*2*+^ of only 0.15, while the cell with Zn@ZnS electrodes displays a high Zn^2+^ conductivity of 0.50. That’s because the hydrated Zn^2+^ moves slower than pure Zn^2+^ in an aqueous solution, and the lower desolvation rate causes the high concentration polarization of Zn (H_2_O)_6_^2+^ near the electrode vicinity. Meanwhile, SO_4_^2−^ anions migrate toward the opposite electrode and result in charge separation, which limits the overall operating voltage because of the building of a junction potential. With the ZnS SEI layer, the decreased diffusion barrier and the fast de-solvation rate lowered the concentration gradient, and a fast Zn^2+^ conductance can be obtained.

The density functional theory (DFT) simulations were further performed to study the interaction between the absorbed Zn atom (adatom) and the Zn or Zn@ZnS electrode. As shown in Fig. 2j, the absorption energy of Zn- Zn@ZnS is − 2.21 eV (ZnS-Zn1 and ZnS-Zn2 represent different adsorption sites, see Fig. S12), much lower than the Zn-Zn interaction (− 0.26 eV), indicating the strong interaction between ZnS and adatom which enables more active sites for Zn^2+^ adsorption. The Bader charge analysis was then calculated to quantitively evaluate the electron transfer upon the adatom adsorption (Figs. 2k and S13). The results indicate with Zn-Zn adsorption, 0.045*e* was transferred from adatom to Zn in the substrate, while 0.180*e* was attracted by S, specifying the preferable and stronger adsorption of the adatom on the ZnS surface. The charge density difference in Figs. 2l and S14 intuitively shows the unbalanced charge distribution across the surface caused by the bonding interaction between the S and Zn adatom. The unbalanced charge distribution accelerates the Zn^2+^ diffusion across the ZnS layer and the deposition process because of the stronger electrostatic attraction toward Zn^2+^ other than the hydrated one in the electrical double layer, advancing the desolvation and reaction rate [31]. For zinc deposition at the Zn@ZnS electrode, there are energy barriers for the Zn^2+^ diffusing through the ZnS SEI layer to the zinc surface, which is 0.035 eV as calculated by DFT (Figs. S15 and S16). Therefore, shortening the diffusion path is required to achieve a fast reaction rate. In this regard, the ultrathin ZnS SEI layer with a thickness of 200 nm can minimize the energy barrier while ensuring a protective effect. As a result, the intrinsic affinity of ZnS toward Zn adatom enables more Zn^2+^ to diffuse through the ZnS film due to the decreased energy barrier and existing abundant nucleation sites, contributing to homogeneous Zn deposition and a dendrite-free morphology. Besides, to study the electrolyte surface in realistic environments, VASPsol was used to study the solvation energy of ZnS and Zn slab in water solvent conditions [45]. The results in Table S1 show that the Zn slab shows negligible solvation energy difference between vacuum and water environments; By contrast, the ZnS slab shows a more negative solvation energy of − 3.694 eV over a supercell of 7.62 × 9.90 Å^2^, implying a higher stabilization of the surface due to solvation [45].

### Suppressed Side Reactions and Zinc Dendrite

**Fig. 3:**
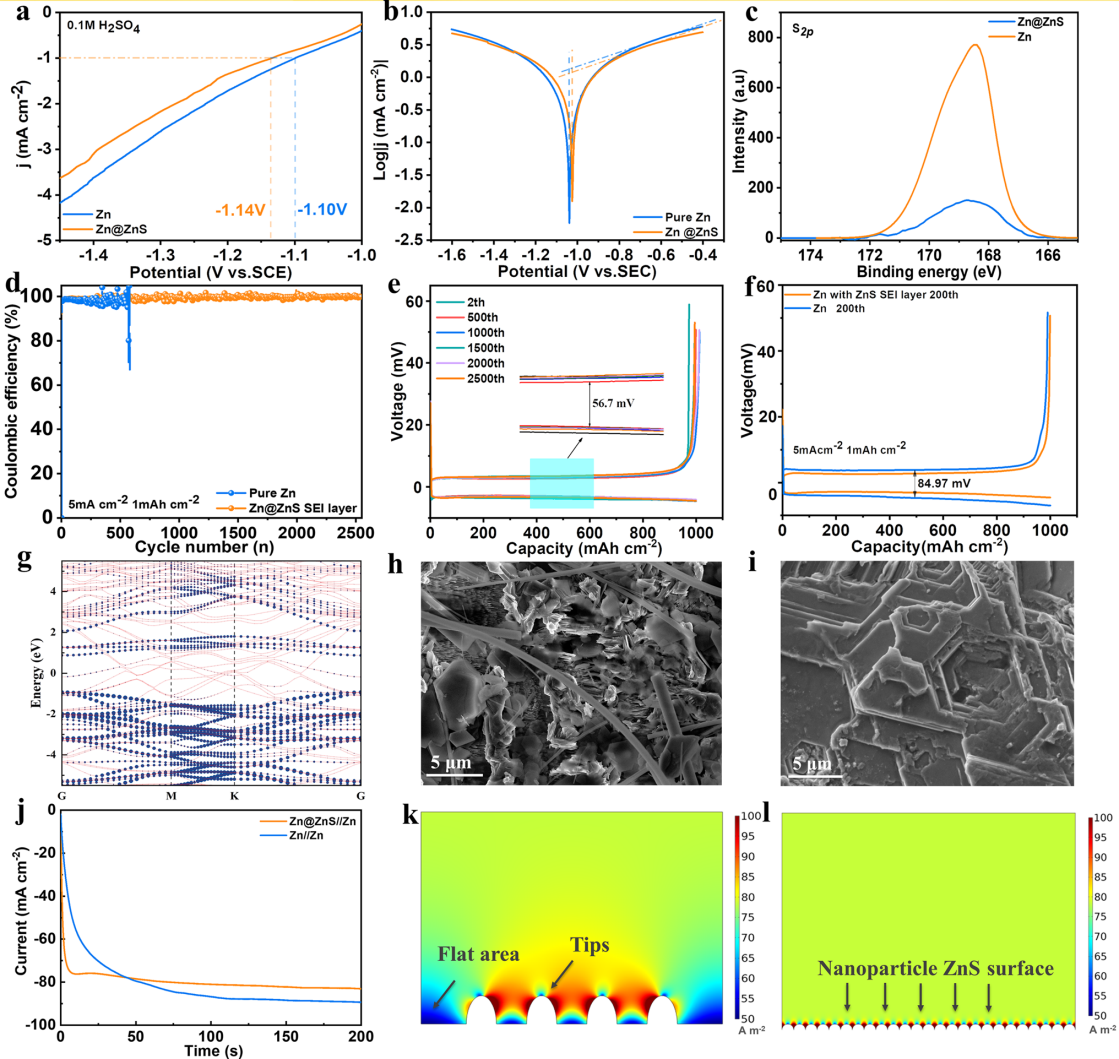
Characterizations of Side Reactions and Zinc Dendrite. **a** LSV curves and **b** Tafel plot. **c** XPS of electrodes after cycling. **d** CE of Zn-Cu cell. **e, f** Charge and discharge curves of **e** Zn@ZnS /Cu and **f** Zn/Cu cell. **g** Band structure of Zn@ZnS interface. **h, i** SEM images of **h** bare Zn surface and **i** Zn@S after 50 cycles at 1 mA cm^−2^ and 1 mAh cm^−2^. **j** CA curves. **k, l** COMSOL simulation of electrolyte current density distribution of **k** the pure Zn and **l** Zn@ZBO during the zinc deposition process

To investigate the impact of regulated EDL on the battery’s performance, electrochemical characterizations were conducted to examine the suppression of side reactions and inhibition of zinc dendrite. The HER suppression was verified by the linear sweep voltammetry (LSV) test (Fig. 3a). The Zn@ZnS electrode requires higher HER overpotential (− 1.14 V *vs.* SCE) than the pure Zn electrode (− 1.0 V *vs.* SCE) for achieving the same reduction current density, indicating the HER is alleviated with the ZnS SEI layer [46]. The relieved zinc corrosion is also evidenced by the Tafel plot (Fig. 3b). With the SEI layer, the corrosion current decreases (1.19 *vs.* 1.33 mA cm^−2^), and corrosion potential increases from − 1.04 to − 1.02 V (*vs.* Ag/AgCl), suggesting the ZnS SEI layer can reduce the corrosion rate. The inhibited side reactions were further confirmed by XPS, X-ray powder diffraction (XRD), and SEM images. The XPS results of Zn@ ZnS after cycling illustrate lower S content at 168.5 eV, corresponding to the S in Zn_4_SO_4_(OH)_6_·H_2_O (Fig. 3c). This implies the formation of the by-product is diminished with the ZnS protection layer, which is consistent with the XRD result and SEM images. Besides, the XPS results of Zn@ ZnS after cycling also show the S 2*p*_1/2_ (162.4 eV) and S 2*p*_3/2_ (161.3 eV) peaks (Fig. S17), which corresponds to the S in ZnS state, indicating the stable existence of ZnS in the cycling process. The XRD results in Fig. S18 display that compared with the bare Zn electrode (0.91), the peak intensity ratio of the by-products and (002) plane (*I*_by-products_/*I*_(002)_) is lower for the Zn@ZnS electrode (0.5), proving limited by-products formation on Zn@ZnS anode after 100 h’s cycling. Figure S19 suggests that after soaking in 2 M ZnSO_4_ (ZSO) for 5 days, a large flake-like by-product appears on the pure zinc surface, which was reported to be the Zn_4_SO_4_(OH)_6_·H_2_O. By contrast, the Zn@ZnS electrode surface shows a dense structure with only a few small flakes observed. These results indicate that the side reactions of HER and Zn corrosion are greatly inhibited with the protection of the ZnS SEI layer.

Coulombic efficiency (CE) was further examined in Zn//Cu asymmetric cells as a key parameter to reflect the reversibility of the battery running. As displayed in Fig. S20 at typical conditions of 1 mA cm^−2^ and 1 mAh cm^−2^, the CE of the cell using pure Zn anode fluctuates and drops in less than 90 cycles; by contrast, the Zn@ZnS||Cu cell cycles more than 220 cycles with an increasing CE, indicating the suppressed side reactions. The reduced voltage polarization (27.3 *vs.* 43.8 mV) and nucleation overpotential (44 *vs.* 56 mV) also confirm the decreased nucleation barrier enhanced reaction kinetics induced by ZnS. Increasing the current density to 5 mA cm^−2^, the cell with Zn@ZnS anode manifests a high CE of 98.9% on average with good stability for more than 2,500 cycles while the CE of Zn||Cu cell fluctuated and failed at ~ 600 cycles (Fig. 3d). Besides, the charge/discharge curves of Zn@ZnS||Cu cell remain stable with a low voltage polarization of 56.7 mV (Fig. 3e), much lower than that using the Zn anode (85.97 mV) (Fig. 3f). This demonstrates that Zn@ZnS could constrain side reactions like the HER and notorious ‘‘dead Zn’’ formation, sustaining a high CE.

The impact of the ZnS SEI layer on the zinc dendrite suppression is theoretically studied by band structure calculation and experimentally verified by SEM images. The band structure of pure Zn, pure ZnS, and Zn@ZnS electrodes is calculated, as shown in Figs. S21 and 3g. The pure Zn metal shows no band gap, while the pure ZnS shows a band gap of 2.07 eV, consistent with the results reported before [47, 48]. Compared with pure ZnS, the Zn@ZnS electrode interface still shows a semiconductor property but with a decreased band gap of 1.81 eV, that is because when the metal and the semiconductor are contacted, electrons from the conduction band of the semiconductor will flow into the metal until both Fermi levels equilibrate. With a semiconductor SEI layer, the zinc ions prefer to deposit under the ZnS film since the interface of Zn and ZnS has a lower potential. This is also proved by our EDX mapping results (Fig. S22). Figure S22 indicates that after 50 cycles, the Zn content in the surface area is lower than that in the bulk area because the zinc ions tend to deposit under the ZnS layer, which is conducive to the uniform deposition of zinc. The regulated zinc deposition with the ZnS SEI layer is further observed via the SEM. As depicted in Fig. 3h, i, the pure zinc surface after 50 cycles shows large flakes that are vertically oriented, potentially leading to the short-circuit of the battery. By contrast, the zinc surface with ZnS thin film presents a flatter surface and horizontally deposited zinc morphology, suggesting a dendrite-free zinc deposition. The uniform deposition of zinc is also confirmed by AFM images depicted in Fig. S23. The pure Zn anode shows a large average roughness (*R*_*a*_) of 159 nm after 25 cycles, whereas a much flatter surface with Ra of 31.1 nm is obtained in the presence of the ZnS SEI layer. The different Zn deposition behavior is further studied by the chronoamperometry (CA) test (Fig. 3j). The classical nucleation theory (CNT) [49] suggests that the zinc nucleus is prone to aggregate during the deposition process to minimize the surface energy through the 2-dimension (2D) diffusion behavior. Then the following Zn^2+^ has a strong propensity to absorb on the preformed tips under the force of a higher electric field, leading to the zinc dendrite growth. Figure 3j shows that with an applied overpotential of − 150 mV, the Zn anode presents a continuous current density increase within 200 s, specifying a rampant 2D diffusion and vertical zinc deposition on the protuberances [50]. By contrast, the response current of the Zn@ZnS electrode stabilized in less than 20 s due to the strong interaction between S^2−^ and Zn^2+^, demonstrating a fast nucleation rate followed by a suppressed diffusion behavior.

To further understand the impact of ZnS-regulated EDL on the behavior of zinc plating, the finite element analysis performed by COMSOL Multiphysics was used to illustrate the zinc deposition behavior [51]. The zinc deposition and growth behavior were simulated at 5 mA cm^–2^. Figure 3k illustrates that the electrolyte current density distribution is highly uneven across the Zn surface, with a significantly higher current near the protuberances than the flat surface area. Additionally, an intense distribution of the electric field can be observed near the protuberances (Fig. S22b). Consequently, the subsequent Zn^2+^ heavily diffuses and deposits around the nucleus pre-formed in the initial stage, leading to the formation of the zinc dendrite. On the contrary, with the SEI layer, the perturbance of the zinc surface was covered by the nanoparticle-like surface and a large surface area can be obtained. As shown in Figs. 3l and S24a, the Zn@ZnS electrode presents a uniform current density and electric field distribution, with a slightly higher current on the boundary of particles, facilitating the uniform Zn deposition and the formation of a flat surface. This is also confirmed by the electrodeposition thickness (Fig. S25). For the Zn electrode, most Zn deposits at the tips and little at the bottom flat area, while the Zn@ZnS electrode shows evenly distributed thickness, demonstrating the ZnS layer can alleviate the dendrite growth and promote uniform zinc deposition.

### Electrochemical Evaluation of Ultra-stable Zn@ZnS Anode

**Fig. 4:**
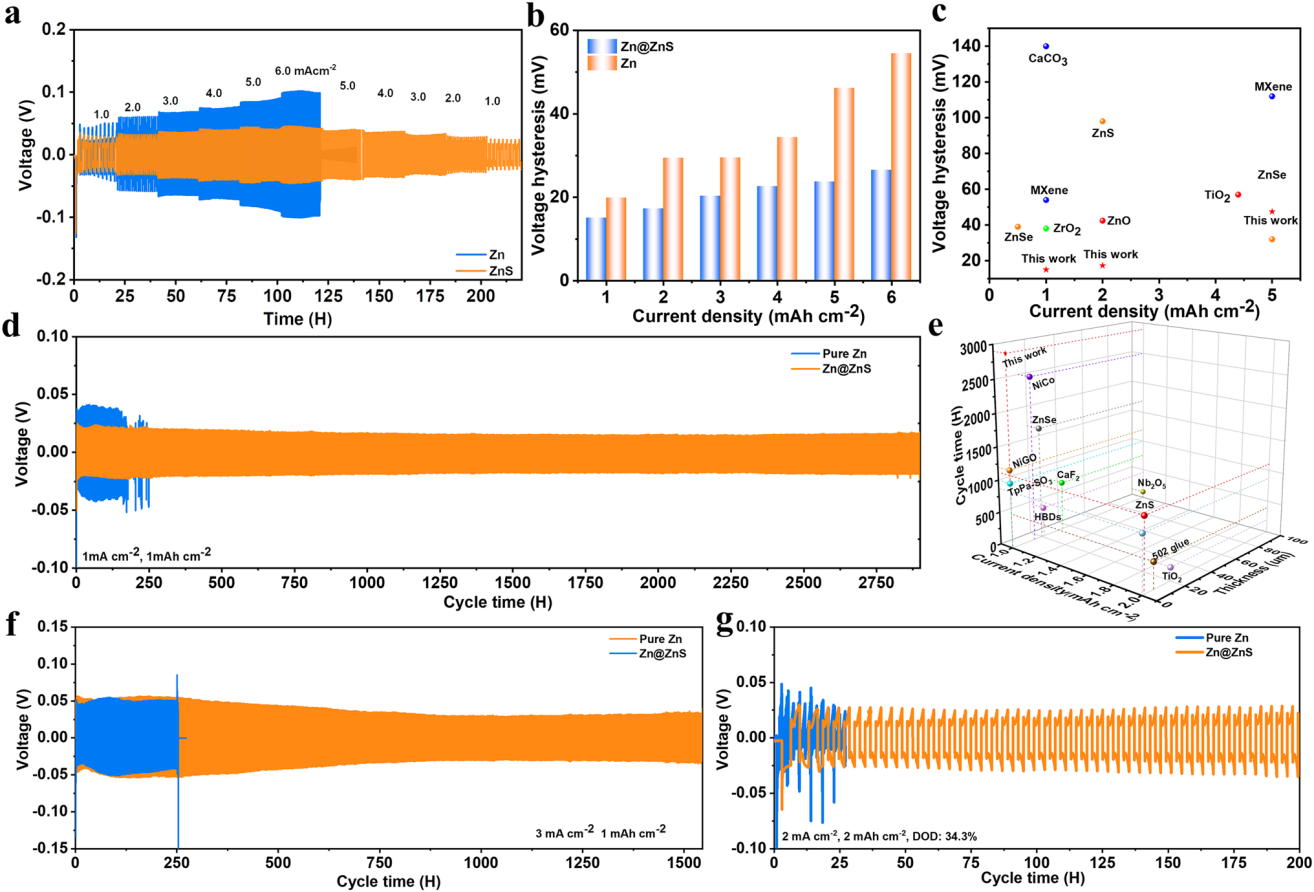
Electrochemical performance of symmetric cells. **a** Rate performance and **b** Voltage hysteresis of symmetric cells. **c** Voltage hysteresis comparison at various current densities. **d** Long cycling performance at 1 mA cm^−2^. **e** Lifetime comparison at various current densities with different coating thicknesses. **f** Long cycling performance at 3 mA cm^−2^. **g** Long cycling performance with a DOD of 34.3%

To further demonstrate the enhanced electrochemical performance of the Zn@ZnS anode, the galvanostatic charging/discharging process was studied in symmetric cells**.** Figure 4a exhibits the rate performance of the cells with a capacity of 1.0 mAh cm^−2^. The cell with Zn@ZnS anode displays excellent cycling stability over various current densities, while the cell using pure Zn anode suffers a short circuit at 6.0 mA cm^−2^. Besides, Fig. 4b indicates the cell with the ZnS protection layer shows much lower voltage hysteresis, especially at higher current density. That is because the good wettability of ZnS film decreases the surface energy and advances the Zn^2+^ diffusion toward the electrode. Besides, the zincophilicity ZnS layer can greatly reduce the diffusion barrier of hydrated zinc ions and promote the desolvation process. Such low voltage hysteresis induced by the ultrathin layer outperforms most previously reported coatings or SEI layers (Fig. 4c), revealing the great potential of the Zn@ZnS anode to achieve fast and deep energy storage.

The long cycling stability of the symmetric cells at various current densities and areal capacities was also carried out to investigate the suppressed zinc dendrite and side reactions induced by the optimized EDL. At typical conditions of 1 mA cm^−2^ and 1 mAh cm^−2^, the Zn@ZnS cell can stably cycle for almost 3,000 h, nearly 15 times longer than that of pure zinc anode (Fig. 4d). Besides, the voltage hysteresis of Zn@ZnS is more stable and much lower than the Zn anode (43.6 vs 81.1 mV) (Fig. S26). With an elevated current density of 3 mA cm^−2^, the battery with Zn@ZnS anode exhibited long cycling for more than 1,500 h, while the battery using pure Zn anode failed after 250 cycles resulting from the internal short circuit caused by zinc dendrite growth (Fig. 4f). At a high current density of 5 mA cm^−2^ and a capacity of 2 mAh cm^−2^ (Fig. S27), the pure Zn cell only cycles for 70 h before the short circuit; By contrast, the battery with Zn@ZnS anode presents a stable running state for more than 350 h along with a lower voltage hysteresis, indicating the faster reaction kinetics and uniform plating of zinc deposits. To evaluate the performance under harsher practical conditions, the cycling performance was studied with a thin zinc foil of 20 μm. At 2 mA cm^−2^ and 0.5 mAh cm^−2^, the battery with ZnS SEI layer can stably cycle for around 600 cycles, in contrast to 150 cycles with pure Zn anode (Fig. S28). At a high depth of discharge (DOD) of 34.3%, the cell with Zn@ZnS anode still sustains a stable performance over 200 h, whereas the cell using pure Zn anode displays a fluctuating voltage and a short working time of 25 cycles (Fig. 4g). The electrochemical performance of the Zn@ZnS electrodes prepared at different reaction times and current densities were also tested at 2 mA cm^−2^ and 2 mAh cm^−2^ (Fig. S29). The results indicate the quality of the ZnS SEI layer largely depends on the charging capacity during the electrodeposition process, and a moderate charging capacity is decisive to getting a ZnS film with good protective effects. For instance, at a low current density of 1.25 mA cm^−2^, the ZnS prepared under a longer deposition time of 8 min shows the best performance, while for a high current density of 5 mA cm^−2^, a shorter deposition time of 1 min achieves the best performance. Overall, the longest lifetime is obtained at a combination of 2.5 mA cm^−2^ and 2 min due to the uniform distribution of nanoparticle ZnS. Figure 4e compares the relationship between the lifetime and coating thickness. Ideally, a long battery life with a thinner coating layer is always desired since the inactive coating could reduce the energy density of the battery. Compared with the previously reported materials, the ultrathin ZnS SEI layer exhibits a competitive advantage in terms of long cycling performance, revealing the great potential of the Zn@ZnS anode to achieve an ultralong dendrite-free deposition behavior [19, 28, 30, 36, 37, 40, 52, 53].

### Electrochemical Performance of Zn@ZnS in Full Cells

**Fig. 5:**
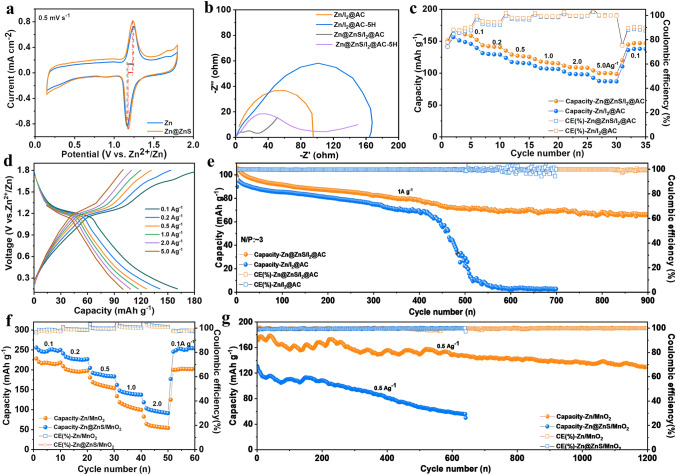
Electrochemical performance of full cells. **a** CV curves, **b** EIS, and **c** Rate performance of Zn//I_2_@AC battery. **d** Galvanic curves of Zn@ZnS //I_2_@AC full cell. **e** Long cycling performance of Zn//I_2_@AC full cell at 1A g^−1^ with N/P ratio of 3. **f** Galvanic curves of Zn@ZnS //MnO_2_ full cells. **g** Long cycling performance of the Zn//MnO_2_ full cells with a current density of 0.5A g^−1^

To test the practical application of the Zn@ZnS anode in ZIBs, the full cells with I_2_/AC and MnO_2_ as cathode materials were assembled. Figure S30a shows the XRD patterns of I_2_ crystal (JCPDS#72–0012), active carbon (AC), and a composite of I_2_/AC. The XRD peaks of the I_2_/AC composite generally match with the XRD peaks of active carbon (AC), both exhibit two broad peaks at around 22° and 43°, corresponding to the (002) and (100) diffraction patterns of amorphous carbon materials. However, no evident I_2_ peaks can be detected in the I_2_/AC, indicating the formation of non-crystalline iodine [54]. Figure S30b indicates the α-MnO_2_ matches with the PDF card of JCPDS#72–1982. Figure 5 shows the electrochemical performance of Zn||I_2_@AC supercapattery and Zn||MnO_2_ battery. The CV curves in Fig. 5a demonstrate that the zinc-iodine supercapattery with Zn@ZnS anode reached a higher current density and smaller redox overpotential due to the faster desolvation process and Zn^2+^ migration rate. This is consistent with the Nyquist plots of the cell at a different resting time shown in Fig. 5b. With the ZnS protection layer, the cells all displayed lower charge transfer resistance at the beginning and after 5 h’ resting, further proving the suppressed side reactions and enhanced interaction of Zn@ZnS anode with Zn^2+^ ions. The galvanostatic charging/discharging process was further conducted to elucidate the impact of the ZnS SEI layer in a full cell. The rate performance in Fig. 5c shows that the full cell with Zn@ZnS anode presents better rate performance and higher specific capacity, and a higher capacity of 160 mAh g^−1^ is reached at 0.1 A g^−1^. The charge and discharge curves of Zn@ZnS||I_2_@AC full cell in Fig. 5d display a wide working window of 0.2–1.8 V and evident working plateaus at ~ 1.2 V, corresponding to the redox potential of I_2_/I^−^ [55]. Figure S31 shows the long-term cycling stability at 10 A g^−1^ of the cells with and without the ZnS layer. As reported, the generation of soluble polyiodide intermediates during the cycling process tends to corrode the zinc anode due to the “shuttle effect” [56]. Herein, to study the influence of the ZnS SEI layer on the inhibition of “shuttle effects”, the cell was subjected to 2,000 cycles followed by a day rest before restarting. Figure S31 shows that during the whole cycling process, the cell with Zn@ZnS anode exhibits higher capacity than the cell using a pure zinc anode. And a high capacity of 115 mAh g^−1^ is obtained in the first 2,000 cycles. After 1 day of rest, the cell can still stably cycle for more than 8,000 cycles, with a high capacity retention of 82%. By contrast, the cell with pure zinc anode exhibits a faster capacity decay after one day’s resting, and the capacity drops to 60 mAh^−1^ after 10,000 cycles. To meet harsh operating conditions, the long cycling performance of full cells with a low N/P ratio of 3 (ultrathin zinc foil of 20 μm) was further studied at a current density of 1A g^−1^ (Fig. 5e). With the ZnS protection layer, the full cell sustains stable cycling for more than 900 cycles, with a capacity retention of 63%. In comparison, the cell with pure zinc anode manifests lower specific capacity than the Zn@ZnS anode in the first 400 cycles and then suffers drastic capacity decay to zero. These results suggest that the side reactions and zinc dendrite are greatly suppressed with ZnS coating.

The electrochemical performances of Zn/MnO_2_ full cells were further studied. The cell with Zn@ZnS anode shows a better rate performance with a higher specific capacity over various current densities (Fig. 5f), and a higher capacity of 250 mAh g^−1^ is obtained at 0.1 A g^−1^, compared to 220 mAh g^−1^ for the bare Zn. This is consistent with the results obtained with I_2_/AC cathode. The higher capacity of full cells in the first few cycles with Zn@ZnS anode can be attributed to the fast reaction kinetics and suppressed side reactions. The charge and discharge curves in Fig. S32 display two typical discharge plateaus caused by consequent H^+^/Zn^2+^ intercalation [57]. Importantly, with Zn@ZnS anode, the cell sustains both higher capacity and long cycling stability of 130 mAh g^−1^ after 1,200 cycles at 0.5 A g^−1^, while the cell with bare Zn under a fast capacity decay within 600 cycles (Fig. 5g). The enhanced performance can be attributed to the following aspects. Firstly, the suppressed side reactions caused by the ZnS SEI layer protect the anode from corrosion and alleviate the cathode dissolution due to the inhibition of water decomposition. Secondly, the good wettability and the enhanced zinc ions migration contribute to the low-concentration polarization and high reactivity in the ZnS-modified battery.

## Conclusion

In this work, we introduced an ultrathin nanoparticle-like ZnS film on the Zn substrate via a facile and controllable electrodeposition method. SEM, DFT, and COMSOL study results verify that this ultrathin ZnS SEI layer with strong adherence to Zn substrate and good wettability is effective in EDL regulation, which provides decreased EDL repulsion and compact zinc deposition. Besides, the electrode with this multifunctional ZnS protective layer shows highly suppressed side reactions of HER and corrosion. Specifically, a high CE of 98.9% and a long cycling hour of 2,500 cycles is achieved in a Zn/Cu symmetric cell at 5 mA cm^−2^, and an ultralong lifetime of around 3,000 h is obtained at 1 mA cm^−2^ and 1mAh cm^−2^. Furthermore, the feasibility of the Zn@ZnS electrode is tested in the Zn||I_2_@AC supercapacitor as well as in the Zn||MnO_2_ battery. When coupled with an I_2_@AC cathode, the cell exhibits stable cycling for more than 10,000 cycles, with a higher capacity and suppressed “shuttle effects.” The Zn||MnO_2_ battery with the ZnS SEI layer also sustains both higher capacity and long cycling stability of 130 mAh g^−1^ after 1,200 cycles at 0.5A g^−1^. This work offers a simple and easily scalable approach for fabricating a highly efficient controllable SEI layer, aimed at regulating the EDL for high-performance zinc-ion batteries.

## Supplementary Information

Below is the link to the electronic supplementary material.

Supplementary file1 (PDF 2785 KB)

## References

[1] S. Wu, Y. Chen, T. Jiao, J. Zhou, J. Cheng et al., An aqueous Zn-ion hybrid supercapacitor with high energy density and ultrastability up to 80000 cycles. Adv. Energy Mater. **9**, 1902915 (2019). 10.1002/AENM.201902915

[2] R. Zhao, J. Yang, X. Han, Y. Wang, Q. Ni et al., Stabilizing Zn metal anodes via cation/anion regulation toward high energy density Zn-ion batteries. Adv. Energy Mater. **13**, 2370034 (2023). 10.1002/aenm.202370034

[3] Q. Jin, J. Xu, Y. Jin, Synergy of regulating zinc electrodeposition and suppressing hydrogen evolution by functional coating layer for highly reversible zinc anode. J. Power. Sources **560**, 232711 (2023). 10.1016/j.jpowsour.2023.232711

[4] H. Cui, L. Ma, Z. Huang, Z. Chen, C. Zhi, Organic materials-based cathode for zinc ion battery. SmartMat **3**, 565–581 (2022). 10.1002/smm2.1110

[5] Y. Lv, M. Zhao, Y. Du, Y. Kang, Y. Xiao et al., Engineering a self-adaptive electric double layer on both electrodes for high-performance zinc metal batteries. Energy Environ. Sci. **15**, 4748–4760 (2022). 10.1039/D2EE02687B

[6] Y. Chen, F. Gong, W. Deng, H. Zhang, X. Wang, Dual-function electrolyte additive enabling simultaneous electrode interface and coordination environment regulation for zinc-ion batteries. Energy Storage Mater. **58**, 20–29 (2023). 10.1016/j.ensm.2023.03.010

[7] L. Ma, J. Vatamanu, N.T. Hahn, T.P. Pollard, O. Borodin et al., Highly reversible Zn metal anode enabled by sustainable hydroxyl chemistry. Proc. Natl. Acad. Sci. U.S.A. **119**, e2121138119 (2022). 10.1073/pnas.2121138119 35675422 10.1073/pnas.2121138119 PMC9214537

[8] Q. Ma, R. Gao, Y. Liu, H. Dou, Y. Zheng et al., Regulation of outer solvation shell toward superior low-temperature aqueous zinc-ion batteries. Adv. Mater. **34**, e2207344 (2022). 10.1002/adma.202207344 36177699 10.1002/adma.202207344

[9] J. Hao, L. Yuan, C. Ye, D. Chao, K. Davey et al., Boosting zinc electrode reversibility in aqueous electrolytes by using low-cost antisolvents. Angew. Chem. Int. Ed. **60**, 7366–7375 (2021). 10.1002/anie.202016531 10.1002/anie.202016531 33440043

[10] H. Jiang, L. Tang, Y. Fu, S. Wang, S.K. Sandstrom et al., Chloride electrolyte enabled practical zinc metal battery with a near-unity Coulombic efficiency. Nat. Sustain. **6**, 806–815 (2023). 10.1038/s41893-023-01092-x

[11] M. Li, X. Wang, J. Hu, J. Zhu, C. Niu et al., Comprehensive H_2_O molecules regulation via deep eutectic solvents for ultra-stable zinc metal anode. Angew. Chem. Int. Ed. **62**, 2215552 (2023). 10.1002/anie.202215552 10.1002/anie.202215552 36536537

[12] R. Chen, C. Zhang, J. Li, Z. Du, F. Guo et al., A hydrated deep eutectic electrolyte with finely-tuned solvation chemistry for high-performance zinc-ion batteries. Energy Environ. Sci. **16**, 2540–2549 (2023). 10.1039/D3EE00462G

[13] J. Cao, D. Zhang, Y. Yue, R. Chanajaree, S. Wang et al., Regulating solvation structure to stabilize zinc anode by fastening the free water molecules with an inorganic colloidal electrolyte. Nano Energy **93**, 106839 (2022). 10.1016/j.nanoen.2021.106839

[14] R. Qin, Y. Wang, M. Zhang, Y. Wang, S. Ding et al., Tuning Zn^2+^ coordination environment to suppress dendrite formation for high-performance Zn-ion batteries. Nano Energy **80**, 105478 (2021). 10.1016/j.nanoen.2020.105478

[15] J. Yang, H. Yan, H. Hao, Y. Song, Y. Li et al., Synergetic modulation on solvation structure and electrode interface enables a highly reversible zinc anode for zinc–iron flow batteries. ACS Energy Lett. **7**, 2331–2339 (2022). 10.1021/acsenergylett.2c00560

[16] X. Peng, T. Li, L. Zhong, J. Lu, Flexible metal–air batteries: an overview. SmartMat **2**, 123–126 (2021). 10.1002/smm2.1044

[17] Y. Lin, Z. Mai, H. Liang, Y. Li, G. Yang et al., Dendrite-free Zn anode enabled by anionic surfactant-induced horizontal growth for highly-stable aqueous Zn-ion pouch cells. Energy Environ. Sci. **16**, 687–697 (2023). 10.1039/D2EE03528F

[18] Y. Lin, Y. Li, Z. Mai, G. Yang, C. Wang, Interfacial regulation via anionic surfactant electrolyte additive promotes stable (002)-textured zinc anodes at high depth of discharge. Adv. Energy Mater. **13**, 2301999 (2023). 10.1002/aenm.202301999

[19] C. Huang, X. Zhao, Y. Hao, Y. Yang, Y. Qian et al., Selection criteria for electrical double layer structure regulators enabling stable Zn metal anodes. Energy Environ. Sci. **16**, 1721–1731 (2023). 10.1039/D3EE00045A

[20] D. Wang, H. Liu, D. Lv, C. Wang, J. Yang et al., Rational screening of artificial solid electrolyte interphases on Zn for ultrahigh-rate and long-life aqueous batteries. Adv. Mater. **35**, e2207908 (2023). 10.1002/adma.202207908 36245304 10.1002/adma.202207908

[21] Y. Yang, C. Liu, Z. Lv, H. Yang, Y. Zhang et al., Synergistic manipulation of Zn^2+^ ion flux and desolvation effect enabled by anodic growth of a 3D ZnF_2_ matrix for long-lifespan and dendrite-free Zn metal anodes. Adv. Mater. **33**, e2007388 (2021). 10.1002/adma.202007388 33554430 10.1002/adma.202007388

[22] Y. An, Y. Tian, K. Zhang, Y. Liu, C. Liu et al., Stable aqueous anode-free zinc batteries enabled by interfacial engineering. Adv. Funct. Mater. **31**, 2101886 (2021). 10.1002/adfm.202101886

[23] D. Xie, Y. Sang, D.-H. Wang, W.-Y. Diao, F.-Y. Tao et al., Frontispiece: ZnF_2_-riched inorganic/organic hybrid SEI: in situ-chemical construction and performance-improving mechanism for aqueous zinc-ion batteries. Angew. Chem. Int. Ed. **62**, 2380762 (2023). 10.1002/anie.202380762 10.1002/anie.202216934 36478517

[24] X. Zhou, P. Cao, A. Wei, A. Zou, H. Ye et al., Driving the interfacial ion-transfer kinetics by mesoporous TiO_2_ spheres for high-performance aqueous Zn-ion batteries. ACS Appl. Mater. Interfaces **13**, 8181–8190 (2021). 10.1021/acsami.0c18433 33560817 10.1021/acsami.0c18433

[25] L. Kang, M. Cui, F. Jiang, Y. Gao, H. Luo et al., Nanoporous CaCO_3_ coatings enabled uniform Zn stripping/plating for long-life zinc rechargeable aqueous batteries. Adv. Energy Mater. **8**, 1801090 (2018). 10.1002/aenm.201801090

[26] Z. Zeng, Y. Zeng, L. Sun, H. Mi, L. Deng et al., Long cyclic stability of acidic aqueous zinc-ion batteries achieved by atomic layer deposition: the effect of the induced orientation growth of the Zn anode. Nanoscale **13**, 12223–12232 (2021). 10.1039/D1NR02620H 34240091 10.1039/d1nr02620h

[27] Y. Cui, Q. Zhao, X. Wu, Z. Wang, R. Qin et al., Quasi-solid single Zn-ion conductor with high conductivity enabling dendrite-free Zn metal anode. Energy Storage Mater. **27**, 1–8 (2020). 10.1016/j.ensm.2020.01.003

[28] Y. Cui, Q. Zhao, X. Wu, X. Chen, J. Yang et al., An interface-bridged organic–inorganic layer that suppresses dendrite formation and side reactions for ultra-long-life aqueous zinc metal anodes. Angew. Chem. Int. Ed. **59**, 16594–16601 (2020). 10.1002/anie.202005472 10.1002/anie.202005472 32519452

[29] W. Shang, Q. Li, F. Jiang, B. Huang, J. Song, B. Zn et al., I2 battery’s performance by coating a zeolite-based cation-exchange protecting layer. Nano-Micro Lett. **14**, 82 (2022). 10.1007/s40820-022-00825-5 10.1007/s40820-022-00825-5 PMC8956761 35334003

[30] M. Cui, Y. Xiao, L. Kang, W. Du, Y. Gao et al., Quasi-isolated Au particles as heterogeneous seeds to guide uniform Zn deposition for aqueous zinc-ion batteries. ACS Appl. Energy Mater. **2**, 6490–6496 (2019). 10.1021/acsaem.9b01063

[31] Q. Lu, C. Liu, Y. Du, X. Wang, L. Ding et al., Uniform Zn deposition achieved by Ag coating for improved aqueous zinc-ion batteries. ACS Appl. Mater. Interfaces **13**, 16869–16875 (2021). 10.1021/acsami.0c22911 33784067 10.1021/acsami.0c22911

[32] K. Ouyang, D. Ma, N. Zhao, Y. Wang, M. Yang et al., A new insight into ultrastable Zn metal batteries enabled by *in situ* built multifunctional metallic interphase. Adv. Funct. Mater. **32**, 2109749 (2022). 10.1002/adfm.202109749

[33] J. Hao, B. Li, X. Li, X. Zeng, S. Zhang et al., An In-depth study of Zn metal surface chemistry for advanced aqueous Zn-ion batteries. Adv. Mater. **32**, e2003021 (2020). 10.1002/adma.202003021 32639067 10.1002/adma.202003021

[34] X. Xie, S. Liang, J. Gao, S. Guo, J. Guo et al., Manipulating the ion-transfer kinetics and interface stability for high-performance zinc metal anodes. Energy Environ. Sci. **13**, 503–510 (2020). 10.1039/C9EE03545A

[35] T.C. Li, Y. Von Lim, X. Xie, X.L. Li, G. Li et al., ZnSe modified zinc metal anodes: toward enhanced zincophilicity and ionic diffusion. Small **17**, e2101728 (2021). 10.1002/smll.202101728 34278715 10.1002/smll.202101728

[36] T. Huang, K. Xu, N. Jia, L. Yang, H. Liu et al., Intrinsic interfacial dynamic engineering of zincophilic microbrushes via regulating Zn deposition for highly reversible aqueous zinc ion battery. Adv. Mater. **35**, e2205206 (2023). 10.1002/adma.202205206 36453716 10.1002/adma.202205206

[37] P. Da, Y. Zheng, Y. Hu, Z. Wu, H. Zhao et al., Synthesis of bandgap-tunable transition metal sulfides through gas-phase cation exchange-induced topological transformation. Angew. Chem. Int. Ed. **62**, 2301802 (2023). 10.1002/anie.202301802 10.1002/anie.202301802 36867435

[38] X. Xu, S. Li, J. Chen, S. Cai, Z. Long et al., Design principles and material engineering of ZnS for optoelectronic devices and catalysis. Adv. Funct. Mater. **28**, 1802029 (2018). 10.1002/adfm.201802029

[39] M. Fayette, H.J. Chang, I.A. Rodrı Guez-Pérez, X. Li, D. Reed, Electrodeposited zinc-based films as anodes for aqueous zinc batteries. ACS Appl. Mater. Interfaces **12**, 42763–42772 (2020). 10.1021/acsami.0c10956 32852196 10.1021/acsami.0c10956

[40] R. Wang, S. Xin, D. Chao, Z. Liu, J. Wan et al., Fast and regulated zinc deposition in a semiconductor substrate toward high-performance aqueous rechargeable batteries. Adv. Funct. Mater. **32**, 2207751 (2022). 10.1002/adfm.202207751

[41] T. Le Manh, E.M. Arce-Estrada, M. Romero-Romo, I. Mejía-Caballero, J. Aldana-González et al., On wetting angles and nucleation energies during the electrochemical nucleation of cobalt onto glassy carbon from a deep eutectic solvent. J. Electrochem. Soc. **164**, D694–D699 (2017). 10.1149/2.1061712jes

[42] K. Ngamlerdpokin, N. Tantavichet, Electrodeposition of nickel–copper alloys to use as a cathode for hydrogen evolution in an alkaline media. Int. J. Hydrog. Energy **39**, 2505–2515 (2014). 10.1016/j.ijhydene.2013.12.013

[43] S. Kumar, S. Pande, P. Verma, Factor effecting electro-deposition process. IJCET **5**, 700–703 (2015). http://inpressco.com/category/ijcet

[44] B. Sarma, R.S. Ray, M. Misra, Charge storage in flower-like ZnS electrochemically deposited on TiO_2_ nanotube. Mater. Lett. **139**, 77–80 (2015). 10.1016/j.matlet.2014.09.115

[45] R. Zhao, H. Wang, H. Du, Y. Yang, Z. Gao et al., Lanthanum nitrate as aqueous electrolyte additive for favourable zinc metal electrodeposition. Nat. Commun. **13**, 3252 (2022). 10.1038/s41467-022-30939-8 35668132 10.1038/s41467-022-30939-8 PMC9170708

[46] G. Li, Regulating mass transport behavior for high-performance lithium metal batteries and fast-charging lithium-ion batteries. Adv. Energy Mater. **11**, 2002891 (2021). 10.1002/aenm.202002891

[47] G. Li, Z. Liu, Q. Huang, Y. Gao, M. Regula et al., Stable metal battery anodes enabled by polyethylenimine sponge hosts by way of electrokinetic effects. Nat. Energy **3**, 1076–1083 (2018). 10.1038/s41560-018-0276-z

[48] T. Altalhi, A. Mezni, M.A. Amin, M.S. Refat, A.A. Gobouri et al., ZnS quantum dots decorated on one-dimensional scaffold of MWCNT/PANI conducting nanocomposite as an anode for enzymatic biofuel cell. Polymers **14**, 1321 (2022). 10.3390/polym14071321 35406194 10.3390/polym14071321 PMC9040719

[49] Q. Zhang, A. Asthagiri, Solvation effects on DFT predictions of ORR activity on metal surfaces. Catal. Today **323**, 35–43 (2019). 10.1016/j.cattod.2018.07.036

[50] H. Qin, W. Kuang, N. Hu, X. Zhong, D. Huang et al., Building metal-molecule interface towards stable and reversible Zn metal anodes for aqueous rechargeable zinc batteries. Adv. Funct. Mater. **32**, 2206695 (2022). 10.1002/adfm.202206695

[51] M. Sharma, D. Mishra, J. Kumar, First-principles study of the structural and electronic properties of bulk ZnS and small Zn_n_S_n_ nanoclusters in the framework of the DFT+U method. Phys. Rev. B **100**, 045151 (2019). 10.1103/physrevb.100.045151

[52] A. Chen, C. Zhao, J. Gao, Z. Guo, X. Lu et al., Multifunctional SEI-like structure coating stabilizing Zn anodes at a large current and capacity. Energy Environ. Sci. **16**, 275–284 (2023). 10.1039/D2EE02931F

[53] C. Ma, X. Wang, W. Lu, C. Wang, H. Yue et al., Achieving stable Zn metal anode via a simple NiCo layered double hydroxides artificial coating for high performance aqueous Zn-ion batteries. Chem. Eng. J. **429**, 132576 (2022). 10.1016/j.cej.2021.132576

[54] Y. Li, S. Yang, H. Du, Y. Liu, X. Wu et al., A stable fluoride-based interphase for a long cycle Zn metal anode in an aqueous zinc ion battery. J. Mater. Chem. A **10**, 14399–14410 (2022). 10.1039/D2TA03550B

[55] Z. Cao, X. Zhu, D. Xu, P. Dong, M.O.L. Chee et al., Eliminating Zn dendrites by commercial cyanoacrylate adhesive for zinc ion battery. Energy Storage Mater. **36**, 132–138 (2021). 10.1016/j.ensm.2020.12.022

[56] J. Zhao, Y. Ying, G. Wang, K. Hu, Y.D. Yuan et al., Covalent organic framework film protected zinc anode for highly stable rechargeable aqueous zinc-ion batteries. Energy Storage Mater. **48**, 82–89 (2022). 10.1016/j.ensm.2022.02.054

[57] S. So, Y.N. Ahn, J. Ko, I.T. Kim, J. Hur, Uniform and oriented zinc deposition induced by artificial Nb_2_O_5_ Layer for highly reversible Zn anode in aqueous zinc ion batteries. Energy Storage Mater. **52**, 40–51 (2022). 10.1016/j.ensm.2022.07.036

