# Supplementary material for: Ultrathin Zincophilic Interphase Regulated Electric Double Layer Enabling Highly Stable Aqueous Zinc-Ion Batteries
Source: Nanomicro Lett. 2024 Jan 25;16:96. doi: 10.1007/s40820-023-01312-1 (PMC10810772; doi:10.1007/s40820-023-01312-1)
Supplement: Supplementary file 1 — Supplementary file1 (PDF 2785 KB) [file 40820_2023_1312_MOESM1_ESM.pdf]

Supporting Information for

Ultrathin Zincophilic Interphase Regulated Electric Double Layer Enabling Highly Stable Aqueous Zinc-Ion Batteries

Yimei Chen[1], Zhiping Deng[1], Yongxiang Sun[1], Yue Li[1], Hao Zhang[1], Ge Li[2], Hongbo Zeng[1,] * and Xiaolei Wang[1,] *

[1] Department of Chemical and Materials Engineering, University of Alberta, 9211-116 Street NW., Edmonton, Alberta T6G 1H9, Canada

[2] Department of Mechanical Engineering, University of Alberta, 9211-116 Street NW., Edmonton, Alberta T6G 1H9, Canada

*Corresponding authors. E-mail: hongbo.zeng@ualberta.ca (Hongbo Zeng), xiaolei.wang@ualberta.ca (Xiaolei Wang)

S1 Experimental Section

S1.1 Material and Methods

Materials: Zinc sulfate heptahydrate ($ZnSO_4·7H_2O$), Manganese sulfate monohydrate ($MnSO_4·H_2O$), iodine, sodium alginate, Dimethylformamide (DMF), and polyvinylidene difluoride (PVDF) were purchased from Thermo Fisher Scientific. Potassium permanganate ($KMnO_4$) and sodium sulfide nonahydrate ($Na_2S·9H_2O$) were purchased from Sigma- Aldrich. Commercial activated carbon (AC, TF-B520) was purchased from MTI Corporation. Sulfuric acid was purchased from Ricca Chemical Company. All the chemicals are in analytic grade and used without further purification.

Electrolyte Preparation: $ZnSO_4·7H_2O$ was dissolved in deionized water to prepare 2M $ZnSO_4$ electrolyte denoted as 2M ZSO. $ZnSO_4·H_2O$ and $MnSO_4·H_2O$ were dissolved to prepare 2M $ZnSO_4$ +0.1M $MnSO_4$ electrolyte.

Electrode preparation: The Zn foil (250 μm, unless otherwise stated) was punched into discs, for the Pure Zn electrode, the zinc foil and then directly used as an anode without treatment. For Zn@ZnS electrode, the two-electrode system was constructed and immersed in 0.2M Na_2S solution. The ZnS film was electrodeposited on the zinc surface galvanostatically. To study the effects of thickness and morphology on the protective performance, the ZnS SEI layer synthesized at various applied currents and deposition time were tested. For I_2/AC cathode, the iodine was melted and diffused into AC at 120 °C for 12 h in the oven, and the final composite consisted of 40 wt% of I_2 and 60 wt% AC. The composite of I_2@AC was mixed with super P and sodium alginate in a mass ratio of 8:1:1 to make the slurry. The slurry was coated on stainless steel mesh (SSM) disk (~1.13 cm^2) and dried in a vacuum oven. Typically, the areal mass loading of I_2@AC was around 3-4 mg cm^{-2}. The α-MnO_2 was synthesized according to previous literature [S1]. $MnSO_4$ (0.507 g, 3 mmol), H_2SO_4 (1 mL,

0.5 M), and 45 mL DI water were put into a beaker and stirred for 10 minutes. Then, $KMnO_4$ (10 mL, 0.1 M) aqueous solution was slowly added to the above solution. The mixture was stirred for 2 h at room temperature. The solution was put into a 100 mL Teflon-contained autoclave and heated at 120 °C for 12 h. The precipitate was washed with DI water and absolute ethanol and then dried at 60 °C overnight. The synthesized MnO_2 was mixed with super P and PVDF in a mass ratio of 7:2:1, and the resulting slurry was coated on SSM and dried under infrared light. The mass loading of α-MnO_2 is 1.5-2.0 mg cm^{-2}.

Electrochemical test: The electrochemical performance of the battery in this paper was tested on Neware battery test system (CT-4008T-5V20mA-164, CT-4008T-5V50mA-164, CT-4008T-5V6A-S1, Shenzhen, China) in coin cells (CR-2032). The rate and long cycling performance of the half-cell were tested in Zn//Zn symmetric cell using 70 µL of electrolyte, while the Coulombic efficiency was conducted ion the Zn//Cu asymmetrical battery with a cut-off voltage of 0.5 V. The Tafel plot was obtained in a three-electrode system with graphite electrode and saturated calomel electrode (SCE) respectively, as the counter electrode and reference electrode. The cyclic voltammetry (CV), electrochemical impedance spectra (EIS), and diffusion curves of chronoamperometry (CA)were conducted on an electrochemical workstation (VMP3, Biologic). The full cell was assembled with Zn anode and cathode materials of I_2@AC or α-MnO_2 which was separated by glass fiber with the addition of 100 µL electrolyte. The performance of the Zn//I_2@AC full cell was measured at a voltage window of 0.15-1.8 V while the Zn// MnO_2 cell was running at 0.15-1.85 V.

Characterization: The crystalline structure of the electrodes was characterized by X-ray diffraction (XRD) conducted on an Ultima IV (Rigaku) diffractometer equipped with a Cu Kα X-ray source. The morphologies of the electrode were observed by scanning electron spectroscopy (SEM, Zeiss EVO M10), filed emission scanning electron spectroscopy (FESEM, Zeiss Sigma) equipped with energy dispersive x-ray analysis (EDX, Oxford). The thickness was tested by an atomic force microscope (AFM) (Dimension Icon, Bruker, USA) in peak-force tapping mode. The surface wettability of the electrolyte was measured by a contact angle measuring system (Dataphysics OCA50).

S1.2 Theory/Calculation

Density function theory (DFT) simulations: DFT simulations were conducted using Vienna Ab initio Simulation Package (VASP) with projector augmented plane-wave method [S2]. For adsorption energy calculation, the generalized gradient approximation was used for the GGA-PBE exchange-correlation functional with a kinetic energy cut-off of 450 eV [S3]. For the Zn adatom -Zn electrode system, four layers of Zn (002) slab with 10.45×10.45 $Å^2$ supercells with a total Zn atoms number of 64 were constructed as substrate. For the Zn adatom -Zn@ZnS electrode system, four layers of ZnS (002) slab with 7.62*9.90 $Å^2$ supercells with a total of 24 Zn atoms and 24 S atoms were constructed as substrate. The bottom two layers of atoms were frozen while the top two were allowed to relax until the force on the atoms was <0.01eV/ Å and the energy difference between the two self-consistent structures was $<10^{-5}$eV. The VASPsol package was used to study the electrode surface in realistic water solvent environments, adopting an implicit model. The Brillouin zone calculations were carried out on a k-point mesh of 3×3×1 for the Zn slab and 4×3×1 for the ZnS slab except for the band

structure calculation, in which a high symmetry KPIONTS is required. The Zn^{2+} diffusion barrier cross ZnS layer was obtained via the climbing image nudged elastic band (Cl-NEB) method. The corresponding initial state, final state, transition state, and other migration structures are shown in Fig. S15. The software Materials Studio (MS) and Vesta were used for model building and result post-processing.

The adsorption energy can be obtained by Eq. (S1):

$$E_{absorption} = E_{total} - E_{Zn} - E_{surface} \quad \text{(S1)}$$

Here $E_{surface}$ and E_{total} are the total energy of the compound before and after Zn adsorption, and E_{Zn} is the energy of a single Zn atom.

The charge density difference can be calculated by Eq. (S2),

$$\Delta\sigma = \sigma(AB) - \sigma(A) - \sigma(B) \quad \text{(S2)}$$

Here AB represents the total energy after the binding between Zn^{2+} and the substrate. A and B are the energy of a single Zn and substrate, respectively.

Finite Element Analysis: COMSOL Multiphysics 5.4 based on Finite Element Analysis was used to simulate the Zn deposition behavior (including the electrolyte current density distribution and deposited zinc thickness) and the electric double layer. For current density distribution and deposited zinc thickness simulation, a simplified 2D model was established, the distance between two electrodes was 15 μm while the length of the electrodes is 20 μm. For pure Zn electrode, the protuberance on the zinc surface was illustrated by semi-ellipses with a short axis of 1 μm and a long axis of 2 μm [S4]. The distance between two semi-ellipses is 4 μm. For Zn@ZnS electrode, a semi-circle with a radius of 0.4 μm was used to simulate the nano-particle-like surface. It's worth noting this model is established on ideal conditions, and cannot fully reflect the real system.

The Secondary Current Distribution was used to solve the electrolyte potential, φ_l (V), according to:

$$i_l = -\sigma_l \nabla \phi_l \quad \text{(S3)}$$

$$\nabla \cdot i_l = 0 \quad \text{(S4)}$$

Here i_l (A m^{-2}) is the electrolyte current density vector and σ_l (S m_{-1}) is the electrolyte conductivity, and the ionic conductivity of 2 M ZSO is set as 1.03 S m^{-1} [S5].

All boundaries except the anode and cathode surfaces are set as insulation:

$$n \cdot i_l = 0 \quad \text{(S5)}$$

Here n is the normal vector, pointing out of the domain.

The reaction that happens on both electrode surfaces is zinc deposition/dissolution,

$$Zn^{2+} + 2e^- \rightleftharpoons Zn \text{ (-0.76 V)} \quad \text{(S6)}$$

The local current density was solved by Butler–Volmer equation:

$$i_{loc,Zn} = i_{0,Zn}(\exp(\frac{\alpha_a F \eta_{Ni}}{RT}) - \exp(\frac{\alpha_c F \eta_{Zn}}{RT})) \quad \text{(S7)}$$

The electrolyte current density on the anode is set to the local current density of the zinc deposition reaction:

$$n \cdot i_l = i_{loc,Zn} \quad \text{(S8)}$$

The model was solved in a time-dependent study, the deposition process lasted for 600 s.

For electric double-layer (EDL) calculation, a simple 1D model (0 - L) representing the electrolyte phase from the electrode surface through the diffuse double layer (EDL) far away to the bulk solution was constructed. The stern layer of the EDL is handled using a boundary condition set at x = 0. The coupled Transport of Diluted Species and Electrostatics physics were used to solve the mass transfer and charge transfer respectively.

The potential was solved according to the Poisson equation:

$$\nabla \cdot (-\varepsilon \nabla \phi) = \rho \quad \text{(S9)}$$

where ε is the permittivity (F/m) and ρ the charge density (C/m^3). The charge density depends on the ion concentrations according to:

$$\rho = F(c_+ - c_-) \quad \text{(S10)}$$

The diffuse layer predicted by Gouy-Chapman theory is in the same order as the Debye length(1/k) for the solution,

$$k^2 = F^2 \sum_i \frac{c_i z_i}{\varepsilon_0 \varepsilon_m RT} \quad \text{(S11)}$$

where ε_m is the solvent relative permittivity.

For boundary conditions, the outer boundary (x=L) which is the bulk electrolyte potential was set as:

$$\phi = 0 \quad \text{(S12)}$$

$$c_i = c_{0,i} \quad \text{(S13)}$$

The stern layer potential is calculated by:

$$n \cdot (-\varepsilon \nabla \phi) = -\frac{\varepsilon \phi_\Delta}{\lambda_S} \quad \text{(S14)}$$

$$\phi_\Delta = \phi_M - \phi \quad \text{(S15)}$$

Φ_M (V) is the applied potential of the electrode measure against bulk electrolyte. The value referred to the experimental result. The Surface Charge Density condition in Electrostatics is

used to define the above condition.

S2 Supplementary Figures

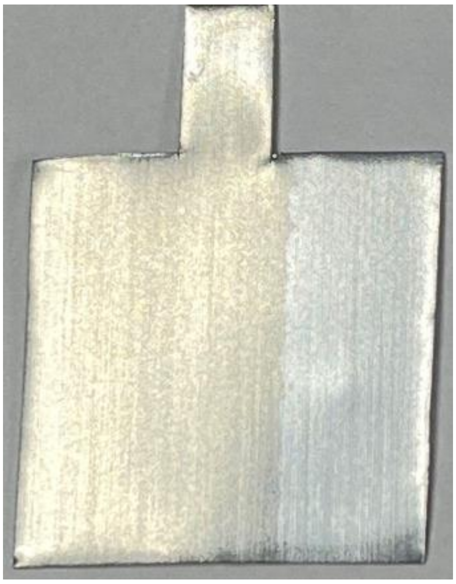

Fig. S1 Zn electrode with (left part) and without (right part) ZnS deposition

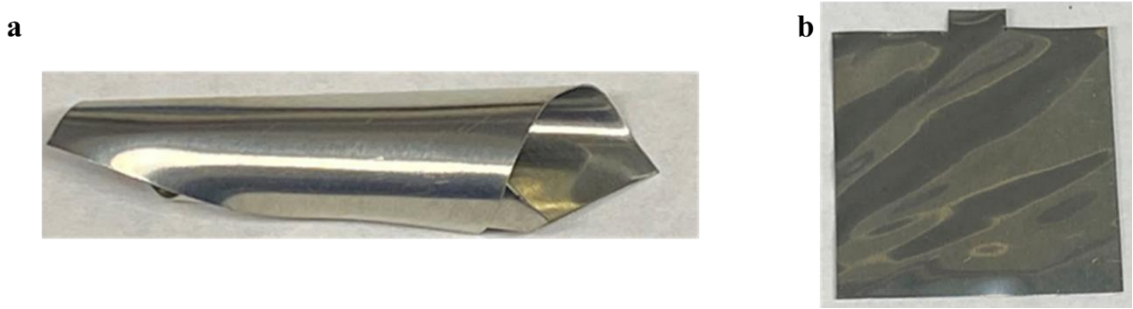

Fig. S2 Optical images of ZnS@Zn at **a** twisting states, and **b** flat states (Zn foil: 100 μm)

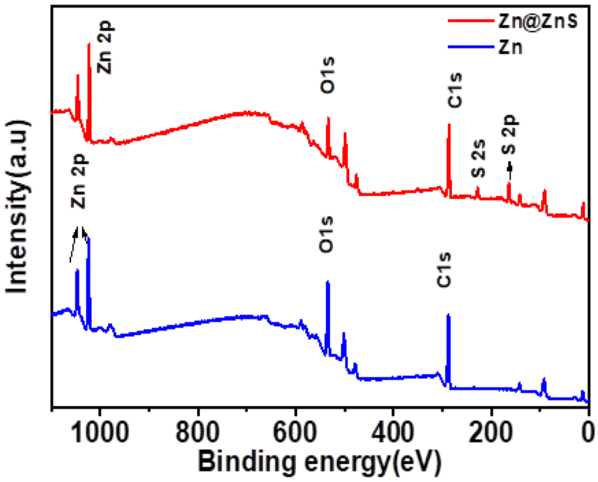

Fig. S3 XPS full spectrum of Zn@ZnS electrode and Zn electrode

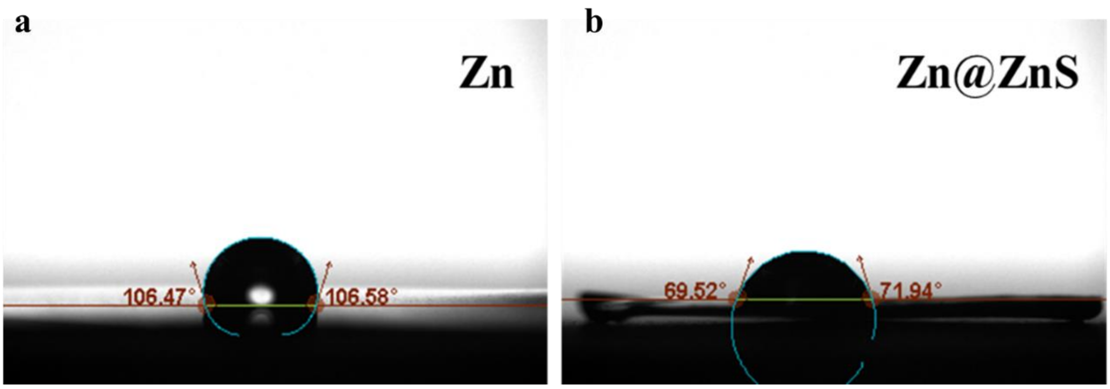

Fig. S4 Contact angle of 2 M $ZnSO_4$ aqueous solution on the Zn foil and Zn@ZnS electrode

According to the Classic Theory of Nucleation (GNT), the Gibbs free energy for the heterogeneous nucleation is given by:

$$\Delta G(n_c) = \Delta G_0 F(\theta) = \frac{16}{3}\frac{\pi\sigma^3 v_M^2}{\Delta\mu^2} F(\theta) \qquad \text{(S16)}$$

Where ΔG_0 is the Gibbs free energy for homogeneous nucleation, and F(θ), is the wetting angle function, given by:

$$F(\theta) = \frac{1}{2} - \frac{3}{4}\cos\theta + \frac{1}{4}\cos^3\theta \qquad \text{(S17)}$$

Lower contact angle, lower Gibbs free energy.

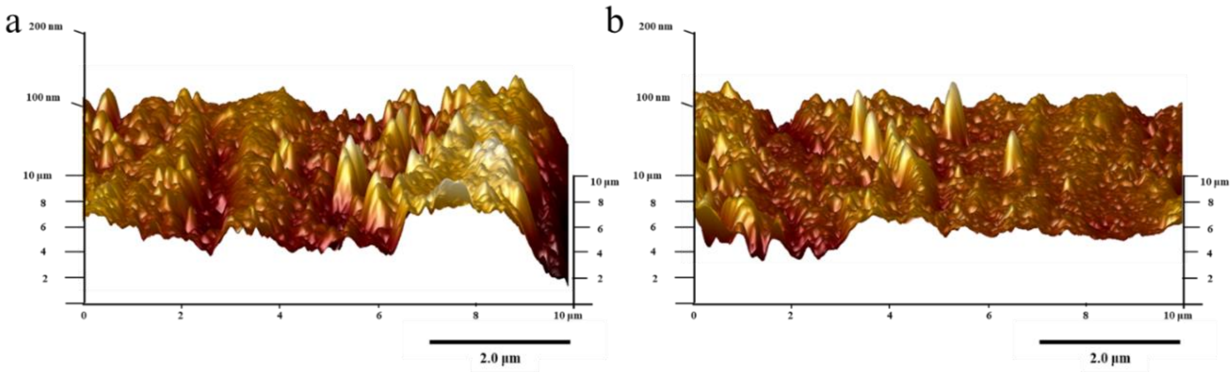

Fig. S5 AFM images of **a** Zn surface and **b** Zn@ZnS electrode surface

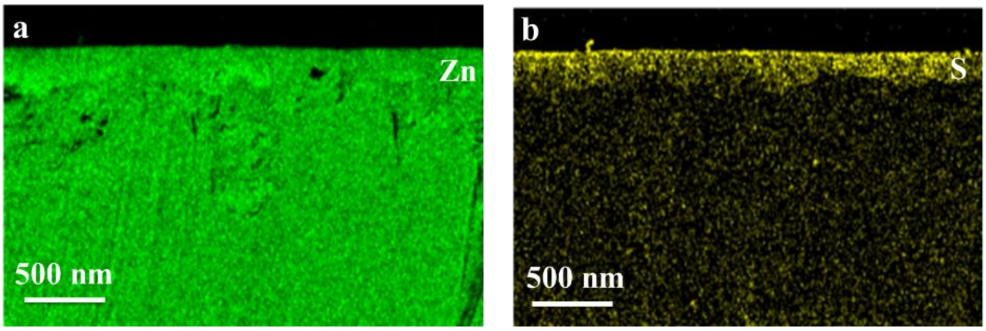

Fig. S6 EDX mapping of cross-section Zn@ZnS electrode

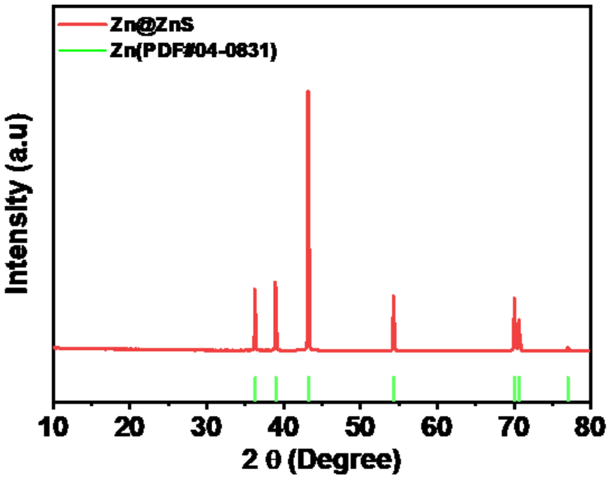

Fig. S7 XRD of prepared Zn@ZnS electrode

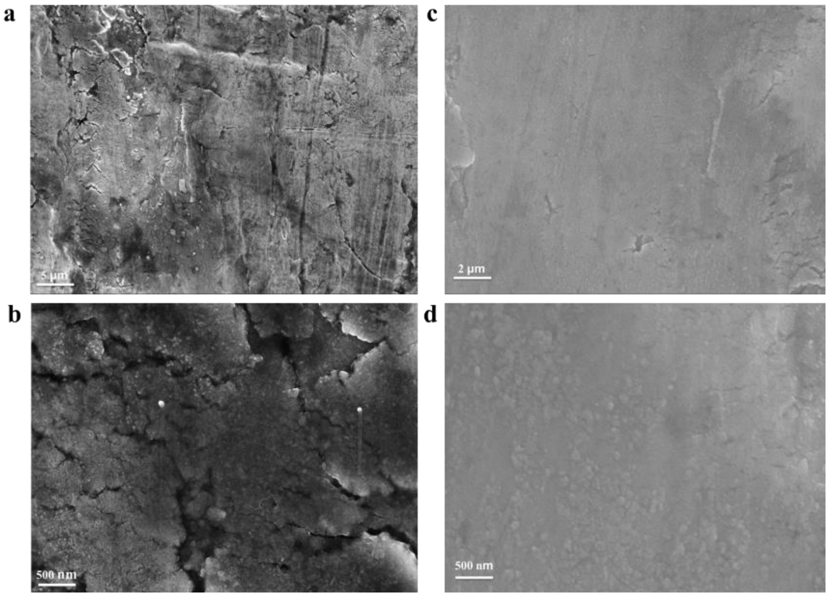

Fig. S8 SEM images of Zn@ZnS electrode electrodeposited at **a, b** 1.25 mA cm^{-2} for 4 min. **c, d** 5 mA cm^{-2} for 1 min

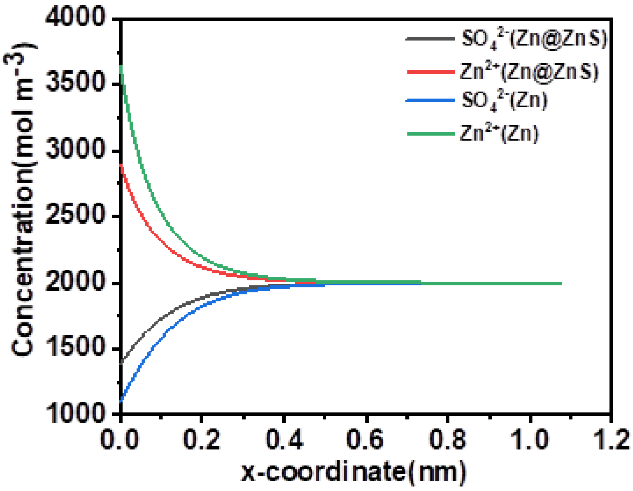

Fig. S9 Concentration gradient of SO_4^{2-} and Zn^{2+} in 2M ZSO solution

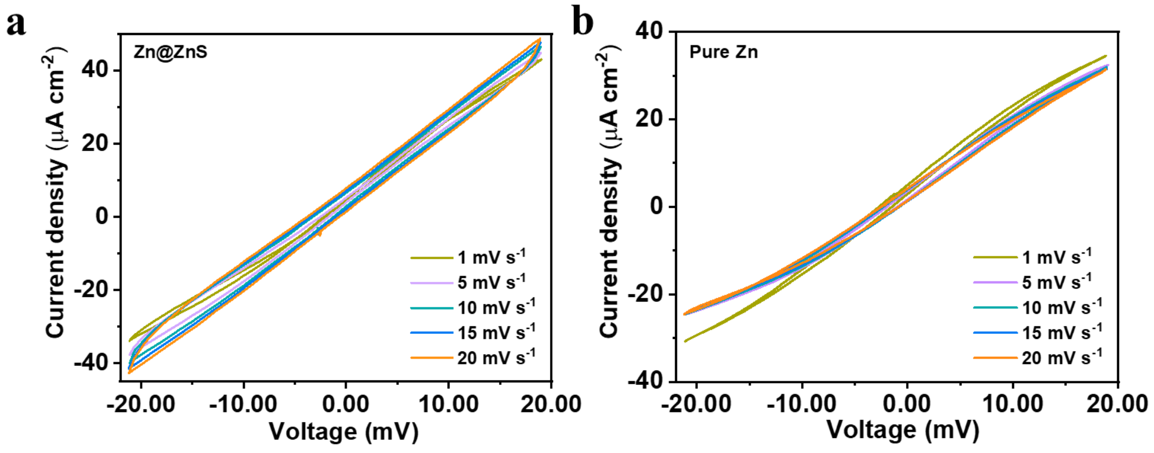

Fig. S10 Electric double layer capacitance measure in **a** Zn symmetric cell and **b** Zn@ZnS symmetric cell

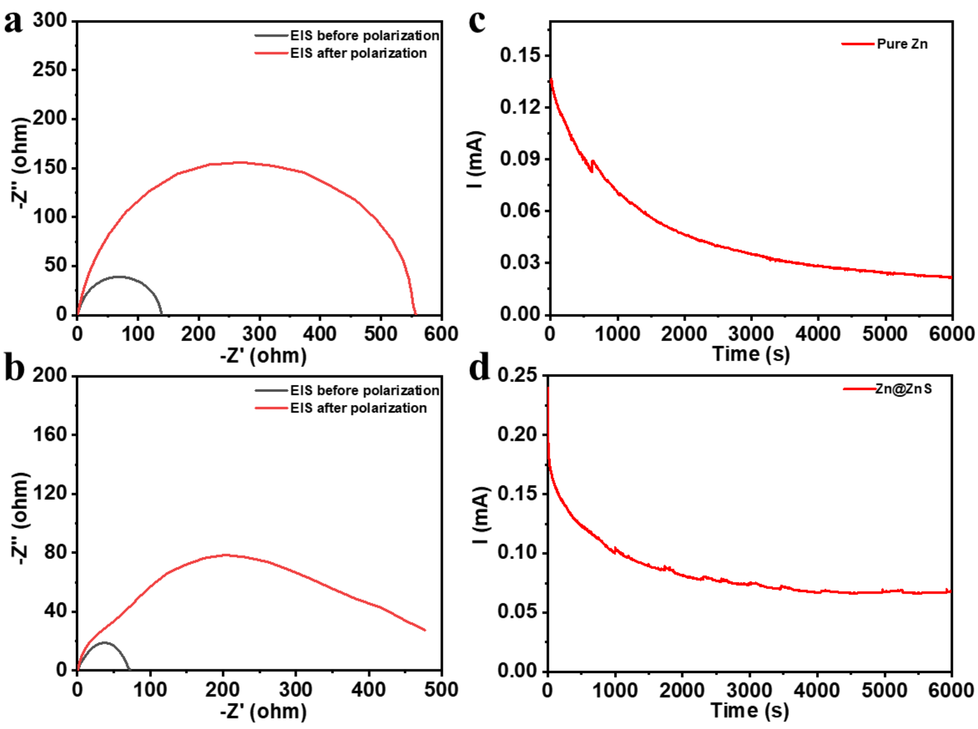

Fig. S11 a, b EIS of **a** Zn and **b** Zn@ZnS before and after polarization at a potential of 25 mV for 6000 s. **c, d** Current-time curves of **c** Zn/Zn and **d** Zn@ZnS/Zn@ZnS symmetric cells

The transference number of Zn^{2+} is given [S6]:

$$t_{Zn^{2+}} = \frac{I_s(\Delta V - I_0 R_0)}{I_0(\Delta V - I_s R_s)} \quad \text{(S18)}$$

Here, ΔV represents the applied voltage; and I_0 and I_s are the initial current and steady-state current, respectively; R_0 and R_s are the initial and steady-state resistance, respectively

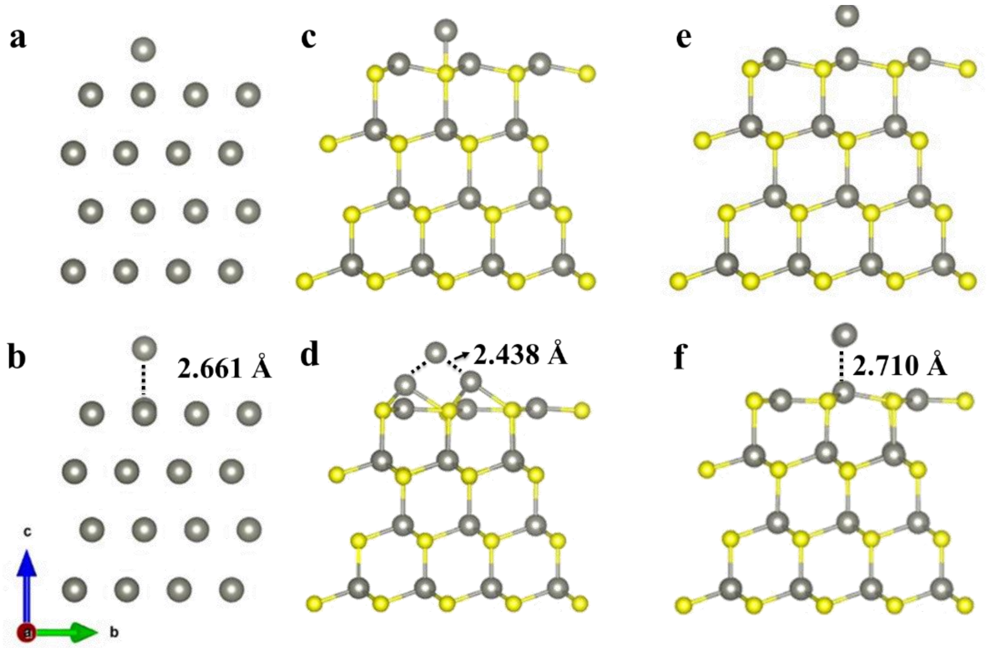

Fig. S12 Adsorption scheme of Zn adatom to **a, b** Zn substrate (**a** original structure and **b** optimized structure). **c, d** ZnS substrate with Zn adatom located above S element (site 1, **c** original structure, and **d** optimized structure). **e, f** ZnS substrate with Zn adatom located above Zn element (site 2, **c** original structure and **d** optimized structure)

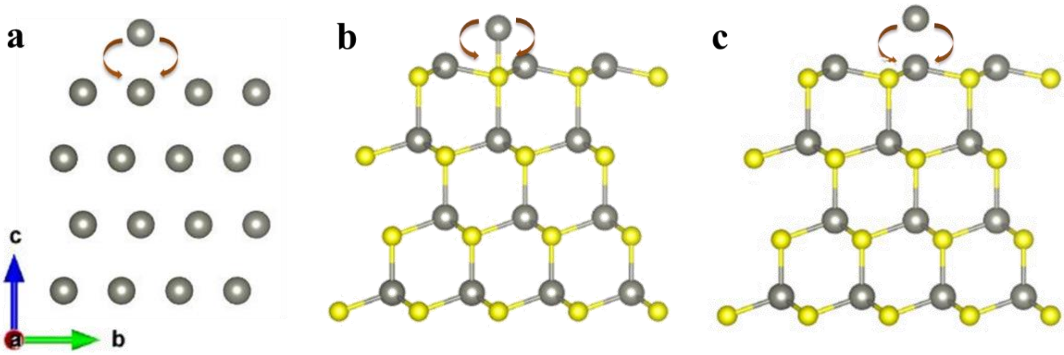

Fig. S13 Bader charge calculation of the original structure of Zn adatom absorbed to **a** Zn substrate **b** ZnS substrate (site 1) **c** ZnS substrate (site 2)

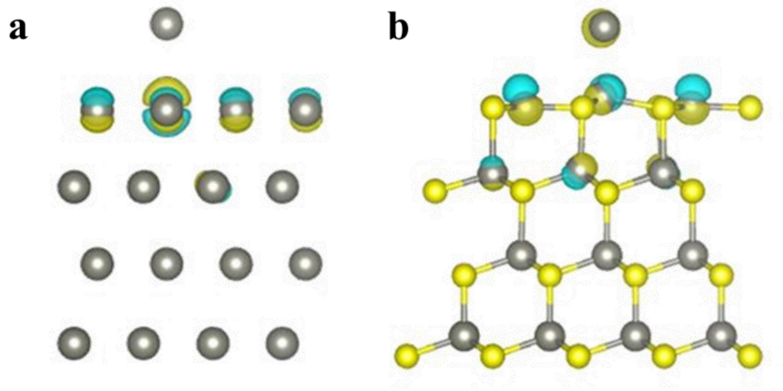

Fig. S14 Charge density difference of Zn adatom absorbed to **a** Zn substrate **b** ZnS substrate (Zn adatom located above Zn atom)

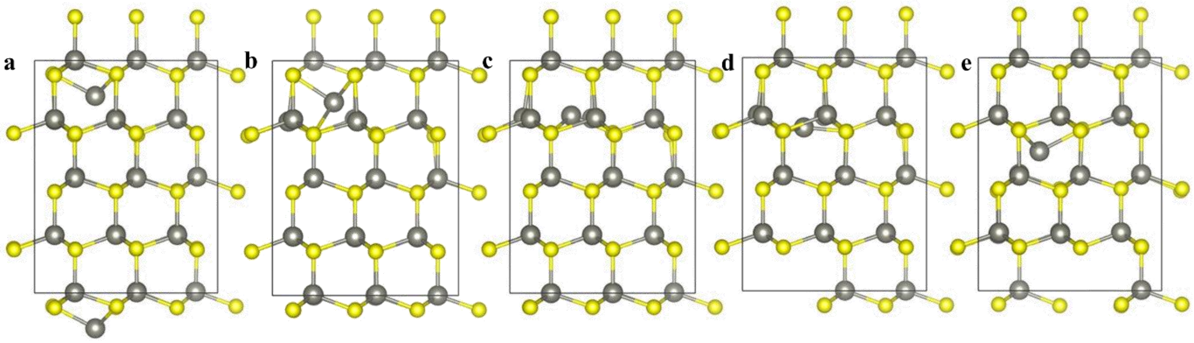

Fig. S15 Diffusion configurations Zn^{2+} through ZnS layer

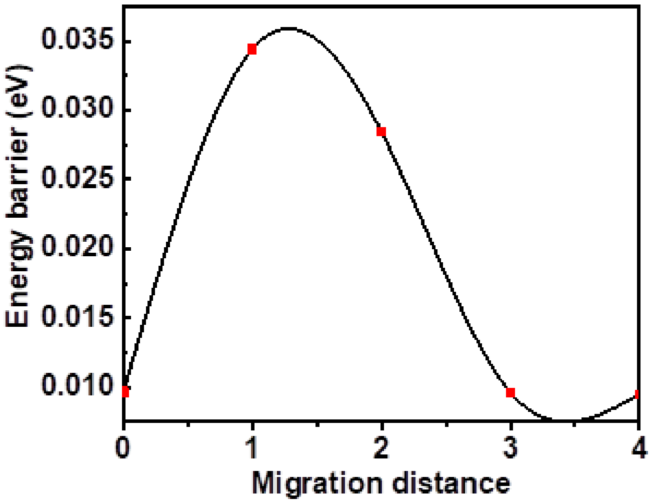

Fig. S16 Diffusion barrier of Zn^{2+} through ZnS layer

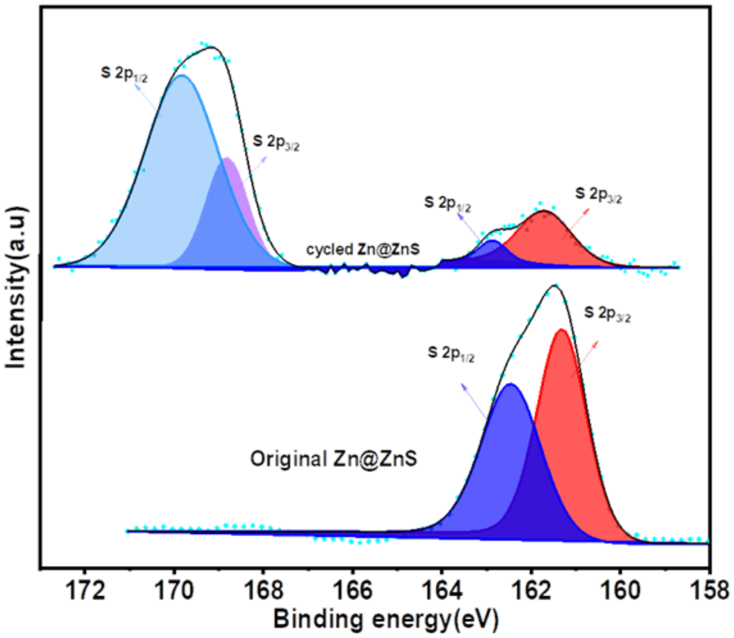

Fig. S17 XPS results of original Zn@ZnS anD Zn@ZnS after 50 cycles

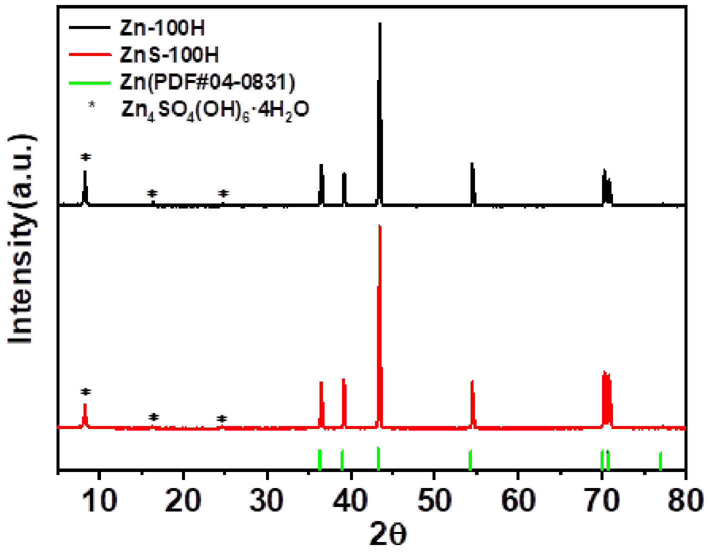

Fig. S18 XRD of Zn and Zn@ZnS electrode after cycling for 100 hours at 1mA cm^{-2} and 1mAh cm^{-2}

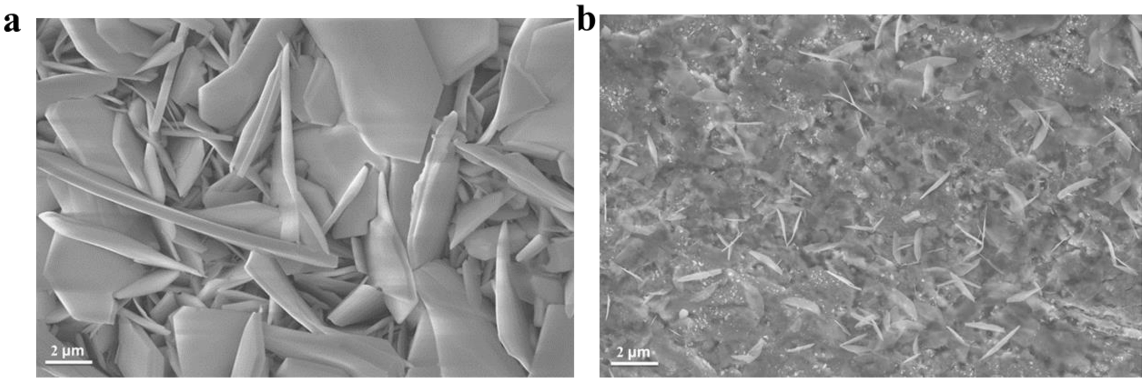

Fig. S19 SEM images of **a** Zn and **b** Zn@ZnS electrodes after soaking in 2 M ZSO for one week

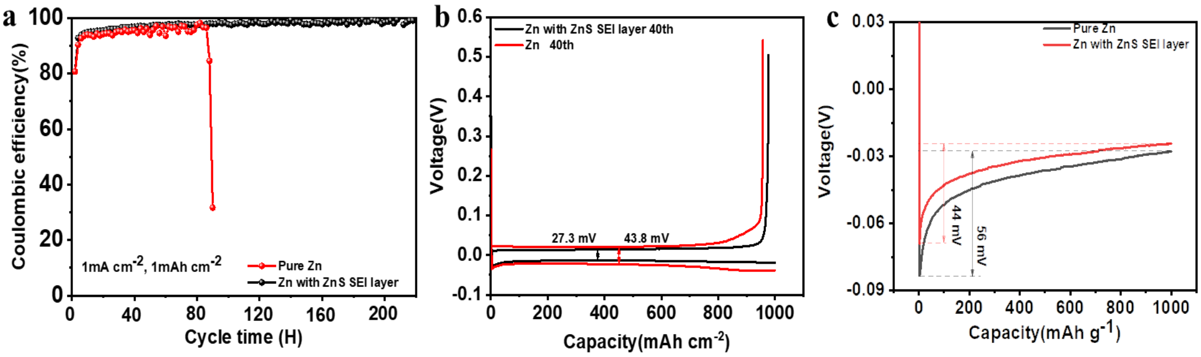

Fig. S20 **a** Coulombic efficiency, **b** polarization overpotential, and **c** nucleation overpotential at 1mA cm^{-2}, 1mAh cm^{-2}

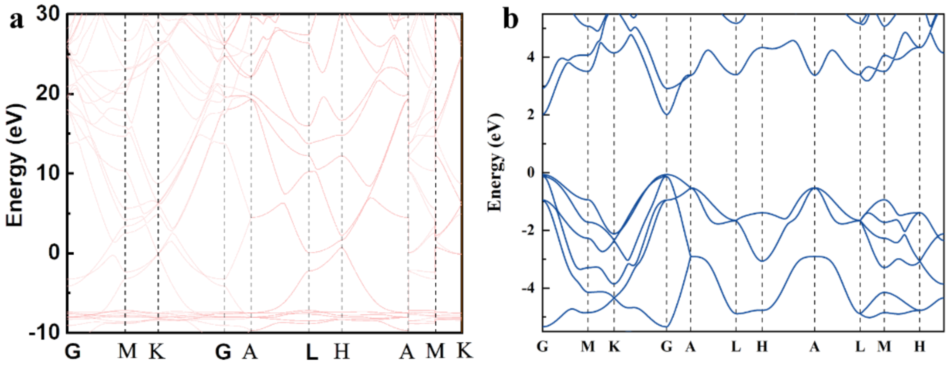

Fig. S21 Band structure of **a** pure Zn electrode and **b** ZnS

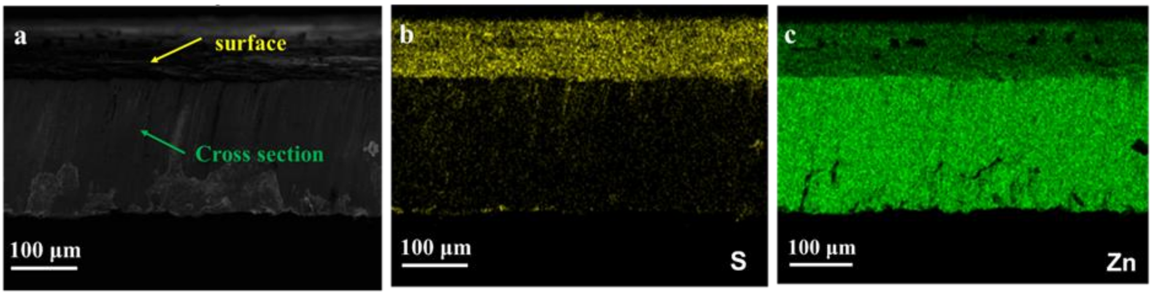

Fig. S22 Cross-section EDX mapping of cycled ZnS electrode

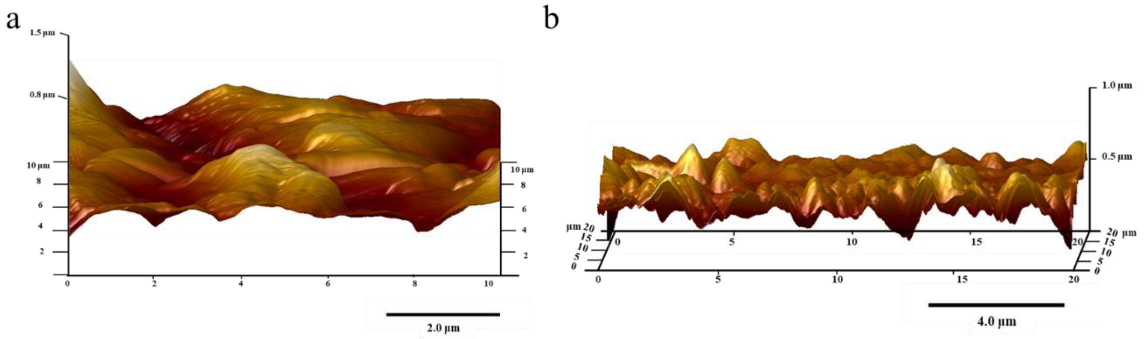

Fig. S23 AFM images of **a** bare Zn surface and **b** Zn@ZnS after 25 cycles at 1 mA cm^{-2}

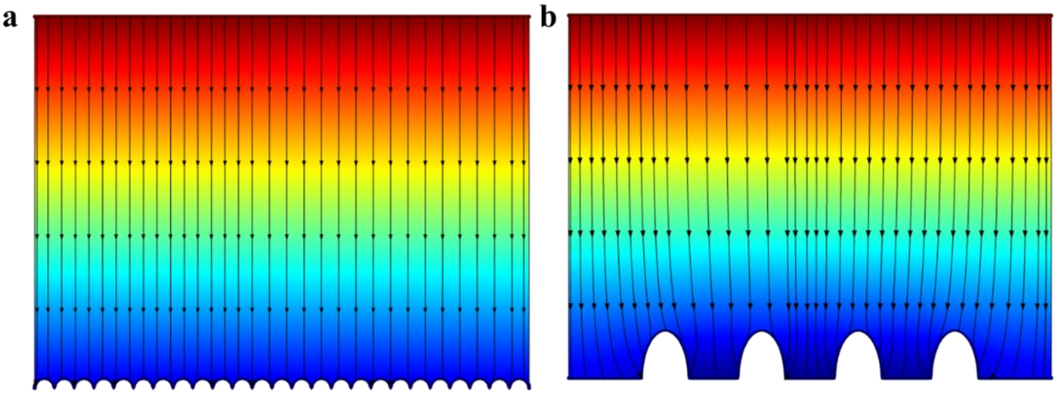

Fig. S24 Electric field distribution of **a** Zn@ZnS electrode **b** Zn electrode

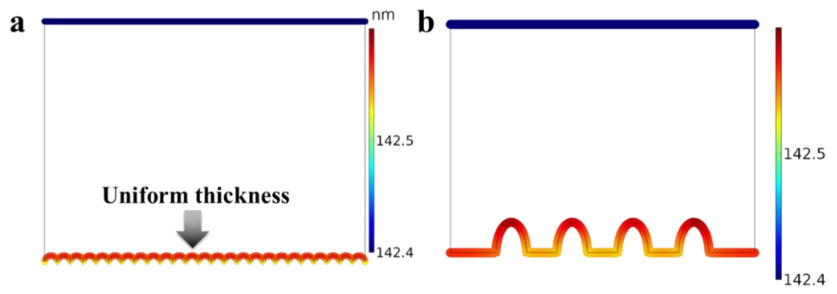

Fig. S25 Electrodeposition thickness after 60 s of **a** Zn@ZnS and **b** bare Zn electrode

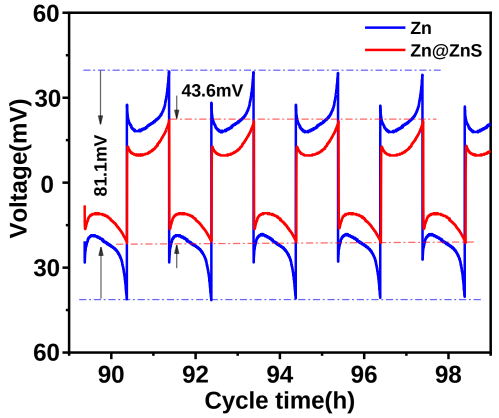

Fig. S26 Voltage hysteresis of Zn and Zn@ZnS symmetric cells at 1mA cm^{-2} and 1mAh cm^{-2}

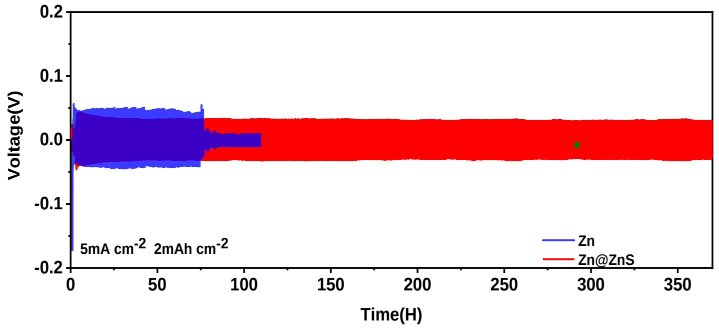

Fig. S27 Long cycling performance of Zn/Zn and Zn@ZnS/ Zn@ZnS symmetric cell at 5 mA cm^{-2} and 2 mAh cm^{-2}

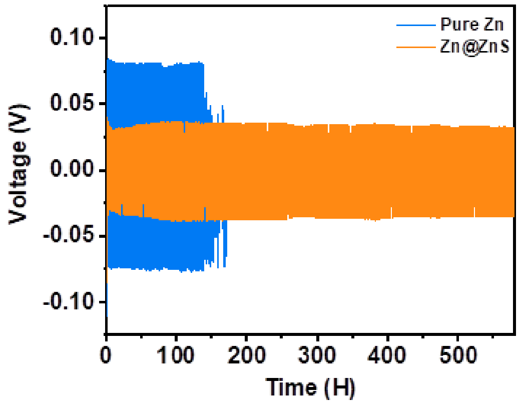

Fig. S28 Long cycling performance of Zn and Zn@ZnS symmetric cells at 2 mA cm^{-2} and 0.5 mAh cm^{-2} with a thin zinc foil of 20 μm

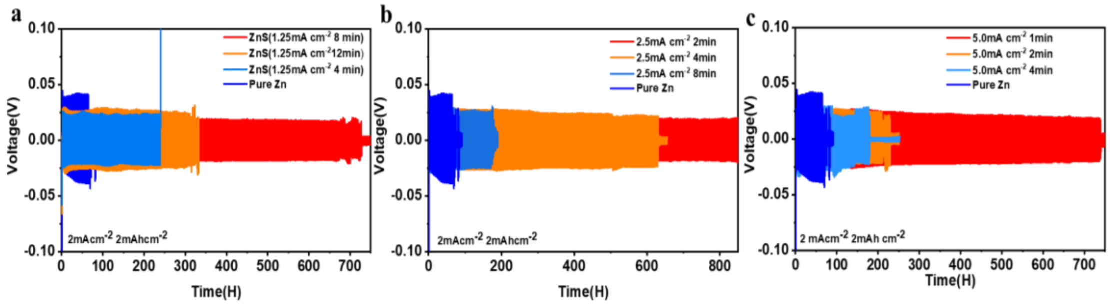

Fig. S29 Long cycling performance of Zn@ZnS/ Zn@ZnS symmetric cells with ZnS electrodeposited at various currents and times

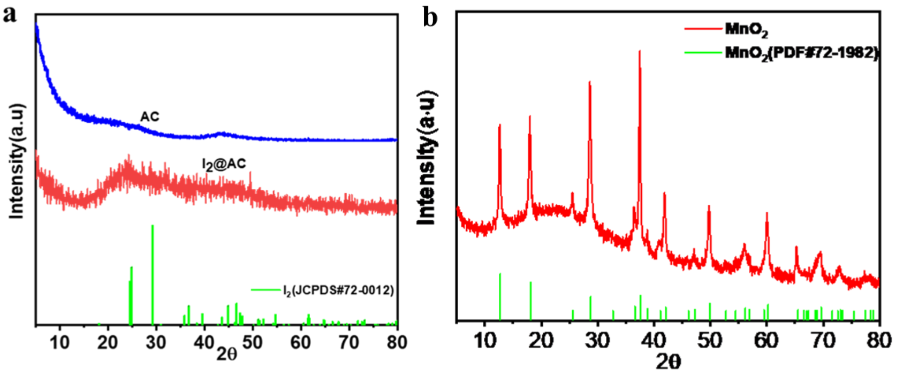

Fig. S30 XRD patterns of **a** I_2@AC and **b** α-MnO_2

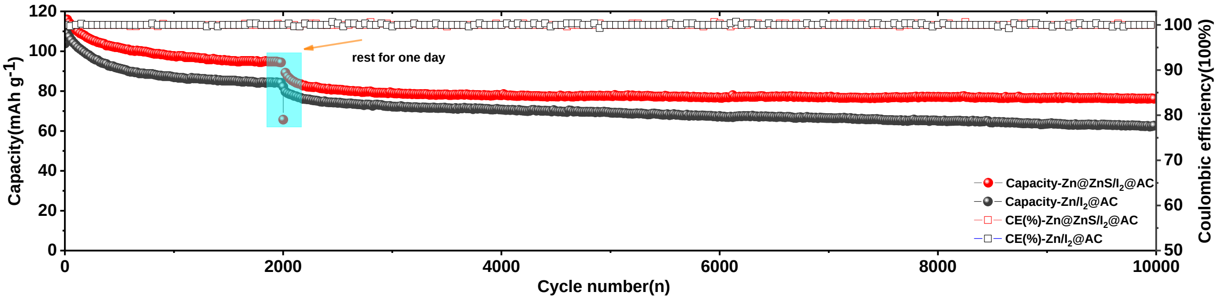

Fig. S31 Long cycling performance of Zn//I_2@AC full cell at 10A g^{-1}

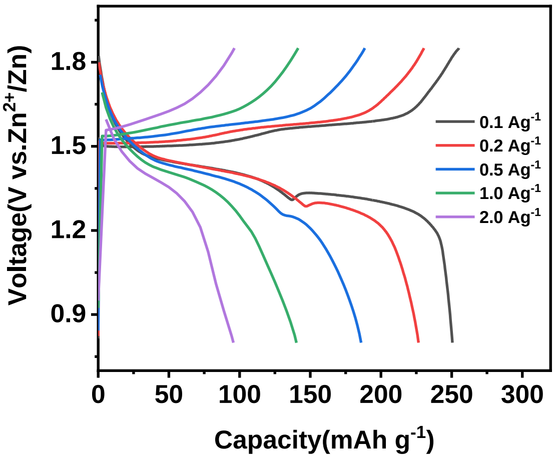

Fig. S32 Charge and discharge curves of Zn//MnO_2 full cells with Zn@ZnS anode

S3 Supplementary Tables

Table S1 Solvation energy comparison of ZnS and Zn surface

	VACUUM	WATER	ΔE
ZnS slab (eV)	-152.884	-156.578	-3.694
Zn Slab (eV)	-67.501	-67.436	0.065

Table S2 EIS fitting results of Zn@ZnS and Zn symmetric cells

	50	60	70	80
R_{ct}_ZnS (Ω)	316.3	226.3	174.4	112.4
R_{SEI}_ZnS (Ω)	60.73	35.73	14.5	13
R_{ct}_Zn (Ω)	987	565	410	260

Supplementary References

[S1]Y. Liao, H.C. Chen, C. Yang, R. Liu, Z. Peng, H. Cao, K. Wang, Unveiling performance evolution mechanisms of MnO_2 polymorphs for durable aqueous zinc-ion batteries, Energy Storage Mater. **44,** 508–516 (2022). https://doi.org/10.1016/J.ENSM.2021.10.039

[S2]G. Kresse, D. Joubert, *From Ultrasoft Pseudopotentials to the Projector Augmented-wave Method*, n.d.

[S3]J.P. Perdew, K. Burke, M. Ernzerhof, *Generalized Gradient Approximation Made Simple*, 1996.

[S4]C. Fu, Y. Wang, C. Lu, S. Zhou, Q. He, Y. Hu, M. Feng, Y. Wan, J. Lin, Y. Zhang, A. Pan, Modulation of hydrogel electrolyte enabling stable zinc metal anode, Energy Storage Mater. **51,** 588–598 (2022). https://doi.org/10.1016/j.ensm.2022.06.034

[S5]S. Zhang, N. Yu, S. Zeng, S. Zhou, M. Chen, J. Di, Q. Li, An adaptive and stable bio-electrolyte for rechargeable Zn-ion batteries, J Mater Chem A Mater. **6,** 12237–12243 (2018). https://doi.org/10.1039/c8ta04298e

[S6]J. Hao, B. Li, X. Li, X. Zeng, S. Zhang, F. Yang, S. Liu, D. Li, C. Wu, Z. Guo, An in-depth study of Zn metal surface chemistry for advanced aqueous Zn-ion batteries, Adv. Mater. **32**, 2003021 (2020). https://doi.org/10.1002/adma.202003021
